# Dopaminergic Radiopharmaceutical Imaging in Parkinsonian Syndromes: From Molecular Targets to Clinical Decision-Making

**DOI:** 10.3390/ph19091369

**Published:** 2026-08-29

**Authors:** Wael Jalloul, Cristina Mariana Uritu, Despina Jalloul, Vlad Ghizdovat, Andreia Vranceanu Ciobanu, Bogdan Ionel Tamba, Cipriana Stefanescu, Irena Cristina Grierosu

**Affiliations:** 1 Department of Biophysics and Medical Physics-Nuclear Medicine, “Grigore T. Popa” University of Medicine and Pharmacy, 700115 Iasi, Romania; 2 Nuclear Medicine Laboratory, “St. Spiridon” County Emergency Hospital, 700111 Iasi, Romania; 3 Advanced Centre for Research and Development in Experimental Medicine “Prof. Ostin C. Mungiu”, “Grigore T. Popa” University of Medicine and Pharmacy, 700259 Iasi, Romania; 4 Department of Pharmacology, Clinical Pharmacology and Algesiology, “Grigore T. Popa” University of Medicine and Pharmacy, 700115 Iasi, Romania

**Keywords:** Parkinson’s disease, parkinsonian syndromes, dopamine transporter (DAT), vesicular monoamine transporter type 2 (VMAT2), [^123^I]ioflupane ([^123^I]FP-CIT), [^18^F]FDOPA, nigrostriatal dysfunction, SPECT, PET, artificial intelligence

## Abstract

Parkinsonian syndromes comprise overlapping neurodegenerative and non-degenerative disorders, making aetiological diagnosis difficult, while existing procedural guidance does not fully integrate tracer-specific biology with clinical decision-making, multimodal strategies, and emerging quantitative and pathology-directed biomarkers. This review synthesises evidence on the molecular targets, radiopharmaceutical characteristics, biological interpretation, and clinical applications of dopaminergic single-photon emission computed tomography (SPECT) and positron emission tomography (PET), focusing on the dopamine transporter (DAT), aromatic L-amino acid decarboxylase (AADC), vesicular monoamine transporter type 2 (VMAT2), dopamine D_2_/D_3_ receptors, differential diagnosis, semiquantification, kinetic modelling, and artificial intelligence-assisted interpretation. The evidence confirms that DAT SPECT with [^123^I]ioflupane ([^123^I]FP-CIT) remains the most established method for demonstrating or arguing against presynaptic nigrostriatal dysfunction, but an abnormal result cannot establish aetiology, and a normal result can redirect evaluation towards non-degenerative mimics; target-specific PET provides complementary biological information, although availability, standardisation, and prospective validation remain limiting. By additionally integrating genetic and prodromal applications, question-driven multimodal imaging, emerging acquisition approaches, α-synuclein imaging and seed amplification assays, and contemporary biological staging frameworks, this review extends procedural guidance and positions dopaminergic imaging as a targeted functional biomarker selected according to the unresolved clinical question rather than as a stand-alone disease label.

## 1. Introduction

Parkinsonian syndromes comprise a heterogeneous group of neurological disorders characterised clinically by bradykinesia in combination with rest tremor, rigidity, or both. Although Parkinson’s disease (PD) is the most common cause of neurodegenerative parkinsonism, similar manifestations may occur in atypical parkinsonian syndromes, including multiple system atrophy (MSA), progressive supranuclear palsy (PSP), and corticobasal syndrome (CBS), as well as in non-degenerative conditions such as essential tremor, drug-induced parkinsonism, functional parkinsonism, and selected forms of vascular parkinsonism. Because clinical features may be subtle, incomplete, or overlapping during the early stages of disease, establishing an accurate aetiological diagnosis may remain challenging even for experienced clinicians [1,2,3].

Presynaptic nigrostriatal dopaminergic dysfunction is a defining feature of PD and a common finding across several degenerative parkinsonian syndromes. Dopaminergic neurons located in the substantia nigra pars compacta project to the striatum, where dopamine modulates basal ganglia circuits involved in the initiation and control of voluntary movement. By the time classical motor manifestations become clinically evident, substantial nigrostriatal dysfunction has generally already developed, limiting the ability of clinical assessment alone to identify early or uncertain disease [2,4].

Molecular imaging enables the in vivo assessment of distinct components of dopaminergic neurotransmission. Relevant targets include the dopamine transporter (DAT), vesicular monoamine transporter type 2 (VMAT2), aromatic L-amino acid decarboxylase (AADC), and postsynaptic dopamine D_2_/D_3_ receptors [2,4,5].

Among currently available techniques, DAT imaging using single-photon emission computed tomography (SPECT) with [^123^I]ioflupane ([^123^I]FP-CIT), commercially available as DaTSCAN^®^ and, in several regions, as generic [^123^I]ioflupane preparations [6,7], remains the most widely implemented method for evaluating presynaptic nigrostriatal dopaminergic integrity in routine clinical practice. Its principal value lies in distinguishing patients with a presynaptic dopaminergic deficit from those with non-degenerative parkinsonian mimics. However, reduced DAT availability is not specific to PD and may also occur in MSA, PSP, CBS, and dementia with Lewy bodies (DLB); therefore, DAT imaging alone cannot reliably determine the precise aetiology of degenerative parkinsonism [3,8,9].

Positron emission tomography (PET) offers complementary approaches by targeting dopamine synthesis, DAT availability, VMAT2 density, or postsynaptic receptor status, with potential advantages in spatial resolution and quantification. Nevertheless, broader clinical implementation remains limited by radiotracer availability, production and distribution logistics, infrastructure requirements, cost, and regulatory approval [4,5,10].

While the 2020 European Association of Nuclear Medicine/Society of Nuclear Medicine and Molecular Imaging (EANM/SNMMI) practice guideline and procedure standard is primarily intended to support the selection, performance, interpretation, and reporting of dopaminergic imaging examinations [3], the present review complements and extends that procedural framework. It provides a clinically oriented, question-driven synthesis of the major dopaminergic SPECT and PET radiopharmaceuticals, linking their molecular targets and tracer characteristics to biological interpretation, clinically relevant limitations, clinical decision-making, and selective multimodal strategies. It additionally integrates evidence published through June 2026 on clinical utility, genetic and prodromal applications, semiquantification and kinetic modelling, artificial intelligence (AI)-assisted interpretation, emerging acquisition and multiparametric approaches, α-synuclein PET and seed amplification assays, and contemporary biological staging frameworks.

## 2. Literature Search Strategy

A narrative literature search was conducted in PubMed/MEDLINE from database inception through June 2026. Search concepts included Parkinson’s disease and parkinsonian syndromes, dopaminergic molecular imaging, dopamine transporter imaging, [^123^I]FP-CIT SPECT, PET radiopharmaceuticals, aromatic L-amino acid decarboxylase, vesicular monoamine transporter type 2, dopamine receptor imaging, semiquantification, artificial intelligence, multimodal imaging, genetic parkinsonism, prodromal synucleinopathy, α-synuclein biomarkers, and biological staging frameworks. Publications were included when they provided directly relevant information on molecular targets, radiopharmaceutical characteristics, image interpretation, diagnostic performance, clinical decision-making, quantitative methods, or multimodal imaging. Guidelines and consensus statements, systematic reviews and meta-analyses, and original clinical studies were prioritised. Seminal earlier publications were retained when required to support neurobiological or methodological concepts, whereas recent studies were preferentially selected when they addressed emerging radiopharmaceuticals, quantitative methods, artificial intelligence-assisted analysis, or multimodal approaches. Study selection was narrative and relevance-based; no formal systematic screening, risk-of-bias assessment, or meta-analysis was performed.

## 3. Neurobiology of the Dopaminergic System

The nigrostriatal pathway originates predominantly from dopaminergic neurons located in the substantia nigra pars compacta (SNpc) and projects to the dorsal striatum, comprising the putamen and caudate nucleus. Dopamine modulates basal ganglia circuits involved in the initiation, selection, and scaling of voluntary movement. Within the canonical model of basal ganglia organisation, dopamine facilitates motor activity by stimulating D_1_ receptor-expressing striatal neurons of the direct pathway and inhibiting D_2_ receptor-expressing neurons of the indirect pathway. Loss of nigrostriatal dopaminergic input disrupts this functional balance and contributes particularly to bradykinesia and rigidity in parkinsonian syndromes [2,11].

Dopamine synthesis begins with the conversion of L-tyrosine into L-3,4-dihydroxyphenylalanine (L-DOPA) by tyrosine hydroxylase (TH), followed by AADC-mediated conversion of L-DOPA into dopamine. Newly synthesised dopamine is transported into synaptic vesicles by VMAT2, which enables regulated storage and release while limiting cytoplasmic exposure to oxidative metabolism. Following vesicular release, dopamine acts on pre- and postsynaptic receptors and is subsequently cleared from the extracellular space predominantly through DAT-mediated reuptake into dopaminergic terminals [4,12].

Nigrostriatal degeneration in PD is topographically heterogeneous. Dopamine depletion is typically most pronounced in the posterior putamen, particularly within its sensorimotor territory, whereas the anterior putamen and caudate nucleus are relatively better preserved during the earlier stages of disease. With disease progression, dopaminergic dysfunction extends to additional striatal regions. This spatial gradient provides the neurobiological basis for the characteristic posterior-to-anterior pattern observed when presynaptic dopaminergic function is assessed in vivo [13].

The molecular components of dopaminergic neurotransmission do not necessarily decline in parallel. During early neurodegeneration, surviving terminals may partially compensate through increased dopamine synthesis and turnover, altered vesicular handling, and reduced reuptake. Multitracer investigations have demonstrated relative preservation or upregulation of AADC activity and a disproportionate reduction in DAT availability when compared with VMAT2 binding [4,14].

This organisation of dopamine synthesis, vesicular storage, receptor signalling, and membrane reuptake provides the biological foundation for targeting different components of dopaminergic neurotransmission with molecular imaging.

## 4. Dopaminergic Radiopharmaceuticals and Molecular Imaging Targets

**Figure 1:**
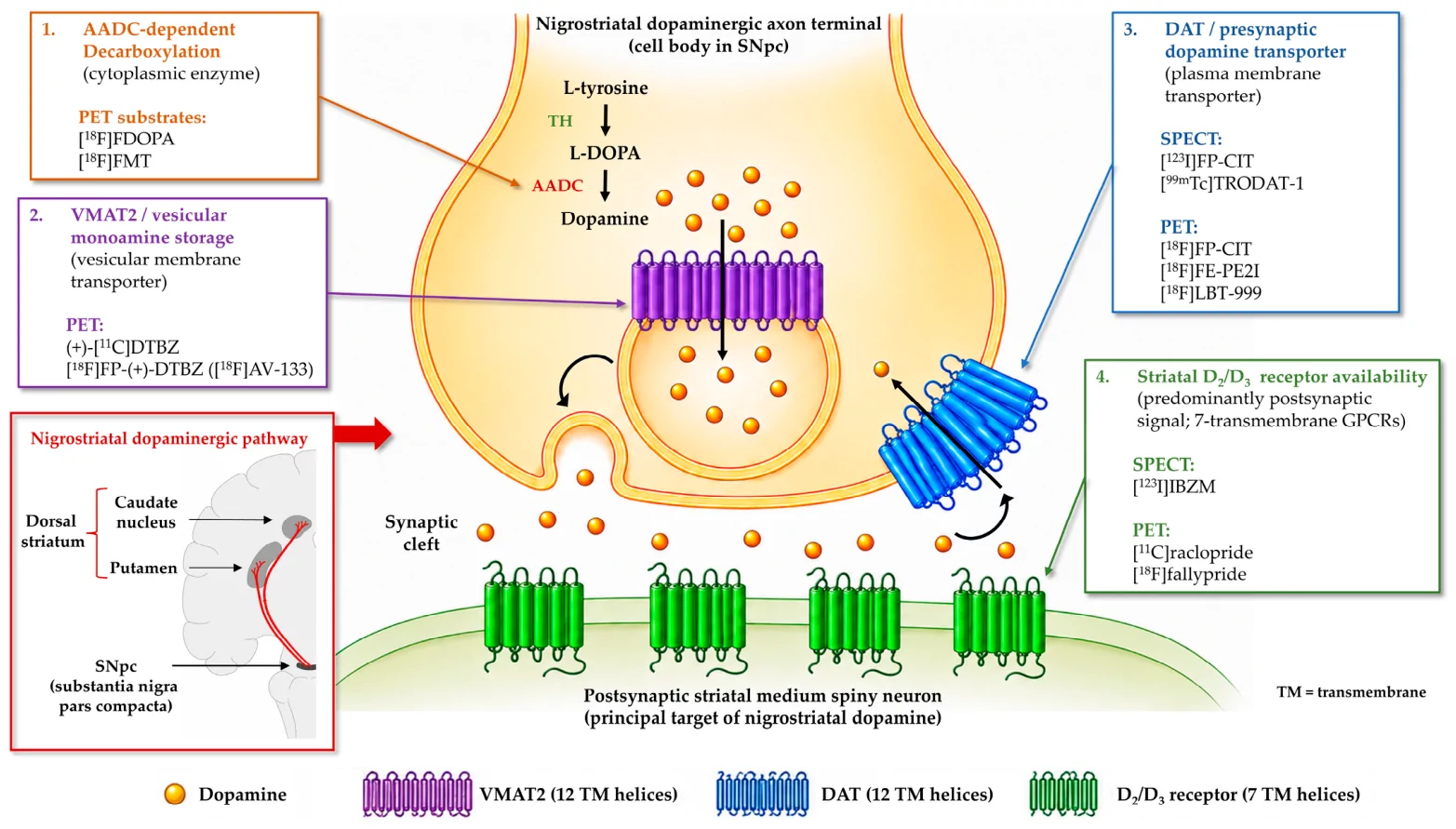
Schematic representation of the nigrostriatal dopaminergic pathway and the principal molecular targets and biochemical processes assessed using dopaminergic imaging. The inset illustrates dopaminergic projections from the substantia nigra pars compacta to the dorsal striatum, comprising the caudate nucleus and putamen. Within the presynaptic terminal, TH catalyses the conversion of L-tyrosine to L-DOPA, AADC converts L-DOPA to dopamine, VMAT2 sequesters cytosolic dopamine into synaptic vesicles, and DAT mediates dopamine reuptake from the synaptic cleft. Dopamine D_2_/D_3_ receptors are represented predominantly on the postsynaptic membrane and mediate dopaminergic signalling. Representative SPECT and PET radiopharmaceuticals are mapped to their corresponding molecular target or biochemical process. DAT and VMAT2 are shown as 12-transmembrane proteins, whereas D_2_/D_3_ receptors are represented as 7-transmembrane G protein-coupled receptors. The diagram is schematic and is not drawn to molecular scale. This is an original figure created by the authors. AADC = aromatic L-amino acid decarboxylase; DAT = dopamine transporter; DTBZ = dihydrotetrabenazine; FDOPA = 6-fluoro-L-DOPA; FMT = 6-fluoro-L-m-tyrosine; GPCR = G protein-coupled receptor; IBZM = iodobenzamide; L-DOPA = L-3,4-dihydroxyphenylalanine; PET = positron emission tomography; SNpc = substantia nigra pars compacta; SPECT = single-photon emission computed tomography; TH = tyrosine hydroxylase; TM = transmembrane; VMAT2 = vesicular monoamine transporter type 2; [^11^C] = carbon-11; [^18^F] = fluorine-18; [^99m^Tc] = technetium-99m; [^123^I] = iodine-123; D_2_/D_3_ = dopamine D_2_/D_3_ receptor subtypes; FP-CIT = ioflupane. AV-133, FE-PE2I, LBT-999, and TRODAT-1 are conventional radiotracer names or development codes.

Dopaminergic radiopharmaceuticals can be classified according to the molecular component of neurotransmission that they target. Presynaptic imaging may assess DAT availability, dopamine synthesis through AADC activity, or vesicular monoamine storage through VMAT2, whereas postsynaptic imaging predominantly evaluates dopamine D_2_/D_3_ receptor availability. These targets differ in their cellular localisation, physiological function, regulation, and susceptibility to compensatory mechanisms. Consequently, the corresponding imaging signals provide complementary rather than interchangeable biological information and should not be interpreted as direct measurements of the absolute number of surviving dopaminergic neurons [2,3,4,5,14,15,16]. The cellular localisation and membrane topology of these targets, together with representative SPECT and PET radiopharmaceuticals, are schematically summarised in Figure 1.

### 4.1. DAT-Targeted SPECT Radiopharmaceuticals

Most SPECT radiopharmaceuticals developed for DAT imaging are tropane derivatives structurally related to cocaine. Their lipophilicity enables passage across the blood–brain barrier (BBB), after which they bind reversibly to DAT located on the plasma membrane of presynaptic dopaminergic axons and terminals, as illustrated in Figure 1. Because DAT is expressed at particularly high density in the caudate nucleus and putamen, specific striatal binding markedly exceeds nonspecific activity in extrastriatal tissue, commonly estimated using the occipital cortex as a reference region. The measured signal therefore reflects the combined effects of cerebral delivery, free and non-specifically bound tracer, radioligand metabolism, binding affinity, and DAT availability [3,4,5,17].

#### 4.1.1. [^123^I]Ioflupane ([^123^I]FP-CIT)

[^123^I]FP-CIT is an N-fluoropropyl derivative of β-CIT. After crossing the BBB, the radioligand binds reversibly to monoamine transporters, with the striatal signal being predominantly determined by DAT because of its high regional expression. Reduced striatal binding is therefore interpreted as evidence of impaired presynaptic nigrostriatal dopaminergic integrity. DAT availability may also be influenced by compensatory regulation, medication exposure, age, sex, and technical factors [3,17,18].

[^123^I]FP-CIT is not completely selective for DAT and also demonstrates affinity for the serotonin transporter (SERT), particularly in extrastriatal regions where DAT density is low. However, the influence of SERT binding on routine striatal assessment is limited because DAT expression predominates within the caudate nucleus and putamen. Compared with its parent compound [^123^I]β-CIT, the fluoropropyl substitution of [^123^I]FP-CIT produces faster cerebral kinetics and earlier development of a stable striatal-to-background contrast, which contributed substantially to its adoption in routine clinical practice [3,17,19].

#### 4.1.2. Earlier and Alternative Radioiodinated DAT Ligands

[^123^I]β-CIT was among the first extensively investigated radioligands for presynaptic monoamine transporter imaging. It has high affinity for DAT but also binds substantially to SERT and the noradrenaline transporter (NET). Its slow association and dissociation kinetics require a prolonged interval before equilibrium-like target-to-background contrast is achieved. Although [^123^I]β-CIT played a fundamental role in establishing DAT imaging as an in vivo marker of nigrostriatal dysfunction, its slower kinetics and limited transporter selectivity reduced its suitability for routine clinical workflows [3,19].

Several second-generation radioiodinated tropane derivatives were subsequently developed to improve transporter selectivity or imaging kinetics. These include [^123^I]IPT, [^123^I]altropane, and [^123^I]PE2I. [^123^I]IPT and [^123^I]altropane enter the brain rapidly and bind to dopamine-rich striatal regions, whereas PE2I was designed to provide greater selectivity for DAT over SERT and NET. Although these radioligands have contributed substantially to the development and validation of DAT imaging, none has achieved the same degree of international regulatory and clinical implementation as [^123^I]FP-CIT [3,17,20].

#### 4.1.3. [^99m^Tc]TRODAT-1

[^99m^Tc]TRODAT-1 combines a tropane-based DAT-binding moiety with a technetium-99m coordination complex. Following passage across the BBB, it binds reversibly to DAT and accumulates preferentially in the striatum in relation to transporter availability. Its principal logistical advantage is the widespread availability of technetium-99m from generator-based systems, reducing dependence on cyclotron-produced iodine-123 and potentially facilitating DAT imaging in regions with limited access to radioiodinated compounds [3,21].

Clinical studies have demonstrated reduced striatal [^99m^Tc]TRODAT-1 binding in PD. Nevertheless, its specific-to-nonspecific contrast and cerebral kinetics are generally considered less favourable than those of [^123^I]FP-CIT. Together with regional differences in manufacturing, regulatory approval, and commercial availability, these characteristics have limited its widespread international adoption, although it remains clinically relevant in selected healthcare systems [3,21].

### 4.2. DAT-Targeted PET Radiopharmaceuticals

DAT-targeted PET radiopharmaceuticals rely on the same biological principle as their SPECT counterparts: passage across the BBB followed by reversible binding to DAT on presynaptic dopaminergic membranes. PET enables higher-sensitivity detection and more detailed kinetic modelling, although interpretation remains dependent on DAT expression, transporter regulation, radioligand selectivity, and endogenous or pharmacological occupancy [4,5,16,22].

#### 4.2.1. [^18^F]FP-CIT

[^18^F]FP-CIT is a fluorine-18-labelled tropane derivative that targets the same presynaptic transporter as [^123^I]FP-CIT. After cerebral delivery, it binds predominantly to DAT within the caudate nucleus and putamen. Its PET signal therefore reflects striatal DAT availability and is subject to the same biological influences affecting other transporter ligands, including presynaptic terminal degeneration, compensatory regulation, and medication-related transporter occupancy [10,23].

Direct comparisons between [^18^F]FP-CIT PET and [^123^I]FP-CIT SPECT have demonstrated broadly concordant patterns of nigrostriatal dysfunction. However, the technical advantages, acquisition characteristics, availability, and comparative clinical performance of the two modalities are considered separately in Section 6 [10,23].

#### 4.2.2. [^18^F]FE-PE2I

[^18^F]FE-PE2I is a fluorinated derivative of PE2I characterised by high selectivity for DAT relative to SERT and NET. Its reversible binding and comparatively rapid kinetics permit assessment of DAT availability in the striatum and, under appropriately validated acquisition and modelling conditions, in selected midbrain regions [4,24].

#### 4.2.3. Other DAT-Targeted PET Radioligands

Several additional DAT PET radioligands have been investigated. Carbon-11-labelled compounds include [^11^C]cocaine (research; historical), [^11^C]methylphenidate (research), [^11^C]CFT (research/specialised-centre), and [^11^C]PE2I (research/specialised-centre). [^11^C]cocaine provided early proof that DAT could be visualised in vivo but has limited transporter selectivity and produces radiolabelled metabolites. [^11^C]methylphenidate has been used in multitracer investigations of compensatory DAT regulation, whereas [^11^C]PE2I provides higher selectivity for DAT and more favourable quantitative characteristics. The short physical half-life of carbon-11 restricts these radioligands mainly to centres with an on-site cyclotron and radiochemistry infrastructure [5,14,16,22,25].

Among fluorine-18-labelled investigational agents, [^18^F]FECNT (research; human evaluation) is a highly DAT-selective tropane derivative with favourable striatal uptake and reversible binding. Initial human studies demonstrated its ability to visualise DAT-rich regions, although radiometabolites and kinetic complexity may influence quantitative measurements. [^18^F]LBT-999 (research; human evaluation) is another fluorinated DAT ligand developed to provide high transporter selectivity, suitable brain kinetics, and regional distribution compatible with quantitative PET imaging. Both remain predominantly research or specialised-centre radiopharmaceuticals [16,22,26,27].

Other fluorinated tropane and phenyltropane analogues have been investigated only at the preclinical stage to improve metabolic stability, transporter selectivity, target-to-background contrast, or kinetic properties. Because most have not progressed to routine clinical implementation, an exhaustive catalogue of preclinical compounds would add limited value to a clinically oriented review [4,5].

### 4.3. AADC-Targeted PET Radiopharmaceuticals

#### 4.3.1. 6-[^18^F]Fluoro-L-DOPA

6-[^18^F]Fluoro-L-DOPA, commonly abbreviated as [^18^F]FDOPA, is a radiolabelled analogue of L-DOPA used to evaluate presynaptic dopamine synthesis and storage. It crosses the BBB through the large neutral amino acid transporter type 1 (LAT1) and is subsequently decarboxylated by AADC to produce [^18^F]fluorodopamine. The radiolabelled amine is then transported into synaptic vesicles by VMAT2, leading to its retention within monoaminergic terminals, as illustrated in Figure 1. The resulting striatal signal therefore depends on transport across the BBB, AADC activity, vesicular sequestration, dopamine turnover, and the functional integrity of surviving presynaptic terminals [3,4,5,14,15,16].

In early neurodegeneration, compensatory increases in AADC activity and dopamine turnover may result in relative preservation of [^18^F]FDOPA uptake compared with DAT-targeted imaging, potentially underestimating the extent of presynaptic terminal degeneration [4,14,15,16].

The interpretation of [^18^F]FDOPA is also influenced by peripheral and central metabolism. Peripheral AADC converts part of the administered compound before it reaches the brain, whereas catechol-O-methyltransferase (COMT) converts [^18^F]FDOPA into 3-O-methyl-[^18^F]FDOPA. This radiolabelled metabolite can cross the BBB and contribute to nonspecific cerebral activity. Pharmacological inhibition of peripheral AADC and, in selected protocols, COMT reduces metabolite formation and improves the relationship between tracer delivery and central dopaminergic trapping [28].

#### 4.3.2. 6-[^18^F]Fluoro-L-m-Tyrosine

6-[^18^F]Fluoro-L-m-tyrosine ([^18^F]FMT) is a non-catecholic substrate of AADC developed for PET evaluation of presynaptic monoaminergic function. Following transport across the BBB, [^18^F]FMT is decarboxylated by AADC to [^18^F]fluorometatyramine. Unlike [^18^F]FDOPA, [^18^F]FMT is not a substrate for COMT and therefore does not generate an equivalent O-methylated radiometabolite. This property reduces one source of nonspecific cerebral activity and may simplify kinetic modelling [16,29,30].

The metabolic trapping of [^18^F]FMT differs from the vesicular retention of [^18^F]fluorodopamine derived from [^18^F]FDOPA. Experimental evidence indicates that [^18^F]fluorometatyramine is poorly retained through VMAT2-mediated vesicular sequestration and undergoes metabolism by monoamine oxidase (MAO), producing a labelled acidic metabolite that contributes to tissue retention. Accordingly, [^18^F]FMT uptake primarily reflects AADC-dependent decarboxylation and subsequent metabolic trapping rather than vesicular dopamine storage. The tracer has remained predominantly a research tool, particularly in investigations of presynaptic dopaminergic function and AADC-targeted gene therapy [16,29,30].

### 4.4. VMAT2-Targeted PET Radiopharmaceuticals

VMAT2 imaging is predominantly performed using radiolabelled derivatives of tetrabenazine. These compounds cross the BBB and bind reversibly to VMAT2 located on the membranes of monoaminergic synaptic vesicles, as illustrated in Figure 1. VMAT2 is expressed in dopaminergic, noradrenergic, serotonergic, and histaminergic neurons and is therefore not intrinsically dopamine-specific. However, because dopaminergic terminals predominate within the striatum, striatal VMAT2 binding mainly reflects the integrity of the nigrostriatal monoaminergic terminal system [4,14,31].

(+)-[^11^C]Dihydrotetrabenazine, abbreviated as (+)-[^11^C]DTBZ, was one of the first PET radioligands used to quantify VMAT2 in the living human brain. The pharmacologically active positive enantiomer demonstrates specific binding to VMAT2, whereas the inactive stereoisomer primarily contributes to nonspecific activity. Reduced striatal (+)-[^11^C]DTBZ binding has been demonstrated in PD, and the ligand has been used as a reference marker in multitracer studies comparing vesicular storage with DAT availability and AADC activity [14,31].

The short physical half-life of carbon-11 motivated the development of fluorine-18-labelled tetrabenazine derivatives. [^18^F]Fluoropropyl-(+)-dihydrotetrabenazine, also known as [^18^F]FP-(+)-DTBZ or [^18^F]AV-133, binds selectively to VMAT2 and demonstrates high striatal retention. Human studies have shown marked reductions in striatal binding in PD, supporting its ability to detect monoaminergic terminal loss [32].

VMAT2 availability is generally considered less susceptible to some short-term compensatory changes than DAT expression or AADC activity and may therefore provide a comparatively stable estimate of presynaptic terminal integrity. However, the observed signal may still be influenced by vesicular biology, ligand stereochemistry, monoaminergic phenotype, medication exposure, and disease-related regulation [4,14,31,32].

### 4.5. Postsynaptic Dopamine Receptor Imaging

Postsynaptic dopaminergic imaging primarily targets D_2_/D_3_ receptors using substituted benzamides or related antagonist radioligands. After crossing the BBB, these compounds bind reversibly to receptors accessible to the tracer. Their measured binding reflects not only receptor density but also receptor affinity state, internalisation, nonspecific binding, medication exposure, and competition with endogenous dopamine. Consequently, changes in radioligand binding cannot automatically be interpreted as proportional changes in the anatomical number of receptors [3,4,33].

[^11^C]raclopride is a relatively low-affinity D_2_/D_3_ receptor antagonist used extensively for PET imaging of striatal receptor availability. Because endogenous dopamine competes with raclopride for receptor binding, increases in extracellular dopamine can reduce apparent radioligand binding. This feature permits investigation of stimulated dopamine release but also complicates comparisons between untreated patients, medicated patients, and different disease stages. Early PD may be associated with preserved or compensatorily increased receptor availability, whereas later disease and prolonged treatment may produce different patterns [33,34].

Higher-affinity PET radioligands, including [^18^F]fallypride and [^18^F]desmethoxyfallypride ([^18^F]DMFP), can evaluate both striatal and lower-density extrastriatal D_2_/D_3_ receptor populations. Their higher affinity improves receptor visualisation but is associated with slower dissociation and more complex kinetic interpretation. [^18^F]DMFP has also been investigated for differentiating idiopathic PD from non-idiopathic parkinsonian syndromes, although postsynaptic receptor imaging has not achieved widespread routine clinical implementation [3,4,35].

For SPECT, [^123^I]iodobenzamide ([^123^I]IBZM) is the most established postsynaptic D_2_/D_3_ receptor radioligand. It binds reversibly to striatal receptors but provides a relatively modest specific-to-nonspecific ratio. [^123^I]epidepride has substantially higher D_2_/D_3_ receptor affinity and permits visualisation of extrastriatal receptor populations. However, its slow kinetics and the methodological complexity of quantifying high-affinity striatal binding have limited routine clinical implementation [3,36].

Imaging of D_1_-like dopamine receptors (D_1_/D_5_) has also been investigated with PET antagonist radioligands, including [^11^C]SCH 23390 and [^11^C]NNC 112, whereas [^11^C]-(+)-PHNO, a D_3_-preferring D_2_/D_3_ agonist radioligand, has been used to investigate the regional contribution of D_3_ receptors. These radioligands remain primarily research tools and have no established role in the routine evaluation of parkinsonian syndromes [4,5].

From a practical radiation-safety perspective, published adult effective-dose coefficients are approximately 0.024 mSv/MBq for [^123^I]FP-CIT, 0.012 mSv/MBq for [^18^F]FP-CIT, 0.023 mSv/MBq for [^18^F]FE-PE2I, and 0.025 mSv/MBq for [^18^F]FDOPA. For an illustrative administered activity of 185 MBq, these coefficients correspond to effective doses of approximately 4.4, 2.2, 4.3, and 4.6 mSv, respectively [37,38,39,40]. These estimates refer to the radiopharmaceutical component alone, exclude any computed tomography (CT) contribution, and may vary with administered activity, dosimetric methodology, hydration, and voiding. Before [^123^I]FP-CIT administration, an appropriate thyroid-blocking agent should be given, such as potassium iodide or Lugol’s solution according to local and product-specific guidance, to reduce thyroid uptake of free iodine-123 [3].

Taken together, dopaminergic radiopharmaceuticals interrogate distinct stages of neurotransmission. [^18^F]FDOPA and [^18^F]FMT assess AADC-dependent biochemical processing through different mechanisms of subsequent retention; DAT ligands evaluate plasma-membrane transporter availability; tetrabenazine derivatives assess vesicular monoamine storage through VMAT2; and dopamine receptor ligands measure receptors accessible to the radiotracer. Selection and interpretation should therefore be guided by the molecular process and clinical question of interest [2,3,4,5,14] (Table 1).

## 5. Clinical Decision-Making in Parkinsonian Syndromes

Building on the molecular targets described above, dopaminergic imaging should be requested to answer a defined and clinically actionable question. In routine practice, the central issue is usually whether presynaptic nigrostriatal dopaminergic dysfunction is present, most commonly assessed using [^123^I]FP-CIT SPECT. The technique is most useful when uncertainty remains between a neurodegenerative parkinsonian syndrome and a condition without presynaptic nigrostriatal degeneration, and when the result is expected to influence diagnosis, management, counselling, or follow-up. An abnormal study supports presynaptic nigrostriatal dysfunction but does not establish its specific aetiology, whereas a technically adequate normal study strongly argues against a clinically relevant presynaptic dopaminergic deficit and should redirect the differential diagnosis [2,3,8,9].

Clinical utility studies support the selective use of DAT imaging in diagnostically uncertain cases. In a prospective multicentre study of 118 patients with clinically uncertain parkinsonian syndromes, the working diagnosis was changed after [^123^I]FP-CIT SPECT in 52% of cases (61/118; calculated 95% confidence interval: 43–61%) [41]. As this study was published in 2004, before the introduction of the 2015 Movement Disorder Society clinical diagnostic criteria for PD, its findings should be interpreted in the context of the diagnostic standards used at that time [1,41]. However, the study population was specifically selected for unresolved diagnostic uncertainty, and the outcome represented a change in the clinician’s working diagnosis following disclosure of the imaging result. The 52% estimate should therefore be interpreted as a measure of decision impact in an enriched referral population rather than as a diagnostic-accuracy estimate or the expected frequency of diagnostic change in unselected patients with parkinsonism.

A subsequent systematic review included 20 studies, of which 13 contributed to each of the two pooled analyses. Changes in clinical management were reported in 54% of patients (95% confidence interval: 47–61%; 13 studies, 950 patients), whereas changes in diagnosis occurred in 31% (95% confidence interval: 22–42%; 13 studies, 779 patients) [42]. Between-study heterogeneity was high for both outcomes (I^2^ = 85% and 96%, respectively), and only four of the 20 included studies were prospective. Patient recruitment and selection criteria, as well as the interval between imaging and clinical reassessment, were frequently incompletely reported, further limiting the generalisability of the pooled estimates [42]. Funnel-plot asymmetry raised the possibility of publication bias or other small-study effects, and preferential publication of studies reporting larger diagnostic or management changes could have inflated the pooled estimates [42,43]. These percentages should therefore be interpreted as context-dependent measures of clinical impact in selected referral populations rather than as uniform effects or estimates of diagnostic accuracy.

### 5.1. From the Clinical Question to the Imaging Decision

The referral question should be formulated in functional rather than disease-specific terms. Instead of asking whether the patient has PD, the clinically appropriate question is usually whether there is evidence of a presynaptic nigrostriatal dopaminergic deficit. This distinction is essential because presynaptic imaging characterises the functional integrity of the nigrostriatal system but does not directly determine the neuropathological disorder responsible for an abnormal result [2,3,4].

Dopaminergic imaging is particularly relevant in patients with isolated or atypical tremor, subtle or inconsistent bradykinesia, equivocal rigidity, uncertain progression, exposure to dopamine receptor-blocking medication, vascular brain disease, functional neurological signs, or cognitive impairment suggestive of DLB. Its contribution is greatest when the clinical findings remain genuinely inconclusive after specialist assessment and when alternative imaging outcomes would lead to different diagnostic or management pathways [3,8,44].

Conversely, imaging usually adds limited value when the phenotype is already typical and confidently classified, when the clinical question cannot be clearly defined, or when the result would not alter management [3,8,9]. Dopaminergic imaging should not be used as a population-screening examination, as a direct measure of disease severity, or as an isolated predictor of levodopa responsiveness or long-term clinical progression [2,3,8].

In healthcare systems where reimbursement pathways require dopaminergic imaging before specialist assessment, a question-driven approach can still be preserved by specifying the unresolved clinical question and applying standardised referral criteria; imaging should support triage and subsequent specialist evaluation rather than be used to assign a stand-alone disease diagnosis.

**Figure 2:**
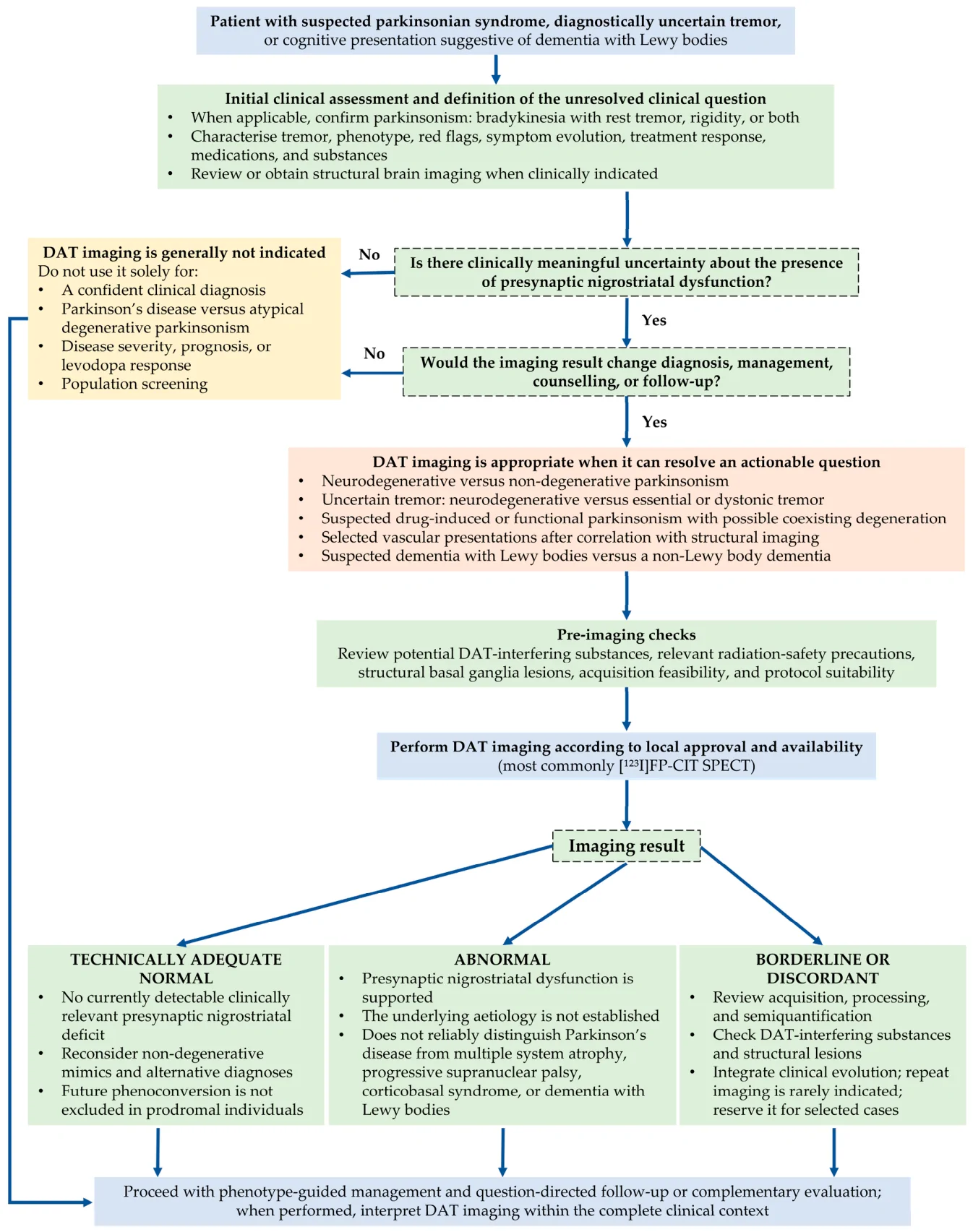
Practical clinical decision algorithm for the selective use of DAT imaging in patients with suspected parkinsonian syndromes, diagnostically uncertain tremor, or relevant cognitive presentations. DAT imaging should be considered when a clinically meaningful uncertainty concerns the presence of presynaptic nigrostriatal dysfunction and when the result is expected to influence diagnosis, management, counselling, or follow-up. An abnormal result supports presynaptic nigrostriatal dysfunction but does not establish its aetiology, whereas a normal result redirects assessment towards alternative diagnoses. Borderline or discordant findings require integrated reassessment of technical quality, semiquantitative measurements, potentially interfering substances, structural imaging, and clinical evolution. [^123^I] = iodine-123; DAT = dopamine transporter; FP-CIT = ioflupane; SPECT = single-photon emission computed tomography.

A practical algorithm integrating the initial clinical assessment, evaluation of whether DAT imaging is appropriate for the unresolved diagnostic question, its anticipated impact on patient management, and interpretation of the imaging findings is presented in Figure 2 [1,3,8,9,44,45].

### 5.2. Degenerative Versus Non-Degenerative Parkinsonism

The principal diagnostic contribution of presynaptic dopaminergic imaging is the distinction between disorders associated with nigrostriatal degeneration and conditions in which presynaptic dopaminergic terminals are expected to remain relatively preserved. Reduced striatal binding increases the likelihood of a neurodegenerative or structurally acquired nigrostriatal disorder, whereas preserved binding redirects the diagnostic process towards alternative causes of parkinsonian or tremulous symptoms [2,3,8,9].

This distinction is particularly useful when clinical signs are mild, incomplete, overlapping, or modified by age, comorbidities, medication exposure, musculoskeletal limitations, or cognitive impairment. The imaging result must nevertheless be interpreted within the context of symptom duration, neurological phenotype, structural imaging, treatment history, and longitudinal evolution rather than treated as an independent diagnosis [2,3,8].

### 5.3. Tremor, Drug-Induced, Functional, and Vascular Parkinsonism

In patients presenting predominantly with tremor, preserved striatal DAT availability favours essential tremor, dystonic tremor, or another disorder without presynaptic nigrostriatal degeneration, whereas reduced binding supports an underlying degenerative dopaminergic process. This distinction is particularly valuable in unilateral, mixed, or atypical tremor when clinical criteria for PD or essential tremor are not yet fulfilled. DAT imaging complements, but does not replace, detailed assessment of tremor phenomenology and longitudinal clinical evolution [3,8,44].

Pure drug-induced parkinsonism caused by postsynaptic dopamine receptor blockade is generally associated with preserved presynaptic DAT availability. An abnormal study in this setting suggests that medication exposure may have unmasked a previously subclinical degenerative parkinsonian disorder rather than being the sole cause of symptoms. Conversely, a normal result supports a non-degenerative mechanism but does not predict the time course or guarantee complete recovery after withdrawal of the causative medication [3,44,46].

Functional parkinsonism is also generally associated with preserved presynaptic dopaminergic imaging when an accompanying neurodegenerative disorder is absent. However, an abnormal study does not exclude a functional component, because functional neurological symptoms and organic nigrostriatal degeneration may coexist. DAT imaging should therefore be used to assess the nigrostriatal system and not as an isolated test to confirm or reject the diagnosis of a functional neurological disorder [3,8,44].

The interpretation of vascular parkinsonism is more complex. Patients with predominantly subcortical white-matter vascular disease may show preserved or only mildly reduced DAT availability, whereas infarcts directly involving the striatum, substantia nigra, or connecting nigrostriatal pathways may produce focal or asymmetric reductions. Structural imaging is therefore indispensable, particularly when defects are sharply demarcated, anatomically confined, or discordant with the expected posterior putaminal gradient of degenerative parkinsonism [3,8,46].

### 5.4. Parkinson’s Disease Versus Atypical Degenerative Parkinsonism

Presynaptic dopaminergic imaging has limited value for differentiating PD from atypical neurodegenerative parkinsonian syndromes. Reduced DAT availability may occur in MSA, PSP, and CBS as well as in PD because each disorder can involve the nigrostriatal system. Although differences in symmetry, caudate involvement, regional gradients, or overall deficit severity have been reported at the group level, their overlap is too extensive for reliable individual aetiological classification [2,3,4,8].

Accordingly, an abnormal study in a patient with early postural instability, recurrent falls, prominent autonomic failure, supranuclear gaze palsy, cerebellar or pyramidal signs, apraxia, cortical sensory loss, or poor levodopa responsiveness should not be interpreted as diagnostic of PD. In such cases, dopaminergic imaging confirms presynaptic nigrostriatal dysfunction, whereas distinction among PD, MSA, PSP, and CBS remains dependent on the clinical phenotype, longitudinal evolution, structural magnetic resonance imaging, metabolic imaging, autonomic evaluation, and other complementary biomarkers [2,3,8].

A normal presynaptic study in a patient initially suspected of having PD should prompt careful reassessment of the diagnosis and structural imaging. Within the Movement Disorder Society clinical diagnostic framework, normal functional imaging of the presynaptic dopaminergic system is listed as an absolute exclusion criterion for PD [1]. However, before applying this criterion, particularly in very early or atypical presentations, the examination should be confirmed as technically adequate and unequivocally normal, as detailed in Section 5.6 [3]. Its application to prodromal individuals is discussed separately in Section 8.1.

### 5.5. Dementia with Lewy Bodies and Cognitive Presentations

Reduced basal ganglia DAT uptake is recognised as an indicative biomarker of DLB. In a patient with dementia and one core clinical feature of DLB, an abnormal presynaptic DAT study can increase diagnostic confidence and contribute to classification as probable DLB. The examination may be particularly helpful when spontaneous parkinsonism is subtle but cognitive fluctuations, recurrent visual hallucinations, rapid eye movement sleep behaviour disorder, or marked sensitivity to antipsychotic medication raise suspicion of Lewy body disease [45].

[^123^I]FP-CIT SPECT has demonstrated clinically useful specificity for differentiating DLB from non-Lewy body dementias, particularly Alzheimer’s disease. However, a normal study does not completely exclude DLB, especially during early disease or when nigrostriatal involvement is limited. DAT imaging should therefore be integrated with the clinical syndrome and other indicative or supportive biomarkers rather than interpreted as an isolated rule-in or rule-out examination [45,47].

DAT imaging does not reliably distinguish DLB from Parkinson’s disease dementia because both conditions commonly demonstrate presynaptic nigrostriatal dysfunction. Their clinical classification depends principally on the temporal relationship between the onset of dementia and established parkinsonism rather than on the distribution or severity of striatal DAT reduction [45,47].

### 5.6. Normal, Abnormal, Borderline, and Discordant Results

A normal presynaptic dopaminergic study indicates that no significant imaging evidence of nigrostriatal dopaminergic deficit was identified at the time of examination. It does not imply that the patient has no neurological disorder. The differential diagnosis may include essential or dystonic tremor, drug-induced parkinsonism, functional parkinsonism, vascular disease without direct nigrostriatal involvement, depression-related psychomotor slowing, musculoskeletal limitations, and other causes of apparent bradykinesia [2,3,8,9].

An abnormal study supports the presence of presynaptic nigrostriatal dysfunction but should not automatically be equated with PD. The differential diagnosis includes PD, MSA, PSP, CBS, DLB, and structural or acquired disorders affecting the nigrostriatal pathways. The report should therefore describe the presence and regional distribution of the deficit while avoiding a specific aetiological conclusion that is not supported by the clinical and structural context [2,3,8].

On visual assessment, a technically adequate normal [^123^I]FP-CIT SPECT examination typically demonstrates bilateral, well-delineated, comma-shaped striatal activity, although mild physiological asymmetry may occur. The pattern classically associated with PD shows predominant loss of posterior putaminal uptake, usually contralateral to the clinically more affected side, with relative preservation of caudate uptake. Conversely, focal “punched-out” defects or reductions spatially corresponding to a basal ganglia lesion should prompt correlation with CT or magnetic resonance imaging (MRI), because infarcts and other structural abnormalities may alter striatal uptake and mimic a degenerative presynaptic deficit [3].

Borderline or clinically discordant findings require reassessment of both the images and the original referral question. Possible explanations include very mild or early dysfunction, physiological asymmetry, age-related decline, motion, head malpositioning, inappropriate reconstruction or reorientation, structural basal ganglia lesions, and incompatibility between semiquantitative measurements and the selected normative database. Visual morphology, semiquantitative analysis, structural imaging, and clinical evolution should be integrated before a definitive interpretation is made [3,8].

The term scans without evidence of dopaminergic deficit (SWEDD) was introduced for patients initially diagnosed with early PD despite presynaptic DAT imaging within normal limits. SWEDD represents a debated and heterogeneous clinical–imaging category rather than a distinct diagnosis. Longitudinal follow-up has generally demonstrated minimal clinical and imaging progression and frequent diagnostic reclassification, suggesting that patients with persistent SWEDD findings are unlikely to have typical idiopathic PD. This interpretation concerns clinically manifest parkinsonism and should be distinguished from normal DAT imaging obtained during a prodromal state, as discussed in Section 8.1 [3,48].

Medicines and recreational substances capable of directly occupying DAT or altering transporter availability may produce misleading reductions in radioligand binding. Relevant agents include cocaine, amphetamine derivatives, methylphenidate, modafinil, bupropion, and selected other centrally acting drugs. Fixed drug-specific washout intervals are not supported by controlled evidence or universal consensus. When discontinuation is clinically safe, current procedural guidance recommends withholding potentially interfering agents for at least five half-lives; however, withdrawal should be individualised because normalisation of DAT binding may take longer in some patients [3,49].

Repeat imaging should not be performed automatically after every normal or equivocal examination. It may be considered when subsequent clinical progression produces convincing new evidence of a degenerative parkinsonian syndrome and when clarification would alter management. Persistent clinical–imaging discordance should prompt reconsideration of the original diagnosis, technical quality, medication exposure, and structural imaging rather than an assumption that either the clinical or imaging assessment must necessarily be incorrect [3,8].

### 5.7. Impact on Diagnosis and Patient Management

Dopaminergic imaging has clinical value when its result is incorporated into a predefined decision pathway. An abnormal examination may increase confidence that presynaptic nigrostriatal dysfunction is present and may influence subsequent therapeutic decisions, counselling, neurological surveillance, and follow-up. Treatment selection should nevertheless remain based on the clinical phenotype, symptom burden, expected benefit, contraindications, and longitudinal treatment response rather than on the imaging result alone [2,3,8,41,42].

A normal examination may prevent prolonged treatment based on an incorrect assumption of presynaptic dopaminergic degeneration and may redirect evaluation towards tremor, dystonia, medication effects, functional neurological disease, vascular pathology, or other alternative diagnoses. However, the decision to discontinue dopaminergic treatment should remain clinical and should consider previous symptomatic response, disease duration, and the possibility of technical or biological discordance [2,3,8,41,42].

Accordingly, the principal clinical contribution of DAT imaging is not simply the visual classification of striatal uptake as normal or abnormal, but its capacity to reduce clearly defined diagnostic uncertainty and redirect the subsequent diagnostic or therapeutic pathway. The magnitude of this contribution depends on patient selection, the referral question, and the way in which imaging results are incorporated into clinical decision-making [41,42].

Changes in diagnosis or management do not necessarily demonstrate improved long-term outcomes. Dopaminergic imaging should therefore be requested selectively, after adequate neurological assessment, and preferably only when the consequences of a normal, abnormal, or equivocal result have been considered in advance. Its contribution is greatest when it resolves a specific clinical question and is integrated with neurological examination, structural imaging, treatment response, and longitudinal follow-up [3,8,42].

An Italian five-year cost-effectiveness Markov model evaluating the differentiation of essential tremor from PD projected that [^123^I]FP-CIT SPECT would provide 1.8 additional years on potentially beneficial therapy and an estimated saving of €442 per patient compared with clinical judgement alone (2005 values); however, these model-based findings are setting- and assumption-dependent [50].

## 6. Comparative and Multimodal Imaging Strategies

No single imaging method provides a complete characterisation of parkinsonian syndromes. Presynaptic dopaminergic imaging determines whether nigrostriatal dysfunction is present, but its ability to establish the underlying aetiology is limited. Structural MRI, cerebral metabolic imaging, postsynaptic receptor imaging, and cardiac sympathetic scintigraphy interrogate different anatomical, functional, and pathophysiological components of disease. These techniques should therefore be selected according to the unresolved clinical question rather than arranged in a fixed hierarchy or regarded as interchangeable diagnostic tests [2,3,4,22].

### 6.1. SPECT Versus PET Imaging of Presynaptic Dopaminergic Function

[^123^I]FP-CIT SPECT remains the most widely implemented method for the clinical assessment of presynaptic nigrostriatal dysfunction because of its extensive validation, established interpretation framework, and comparatively broad availability. DAT-targeted PET radiopharmaceuticals offer higher spatial resolution, greater counting sensitivity, and more flexible quantitative and kinetic modelling. Because both modalities interrogate DAT, they share the aetiological limitations discussed in Section 5.4 [3,4,10,23,24].

Direct comparisons have demonstrated broadly concordant classification of presynaptic dopaminergic deficit using [^18^F]FP-CIT PET and [^123^I]FP-CIT SPECT [23]. Similarly, [^18^F]FE-PE2I PET has shown high concordance with [^123^I]FP-CIT SPECT while offering greater DAT selectivity and the possibility of assessing selected midbrain regions under validated conditions. Nevertheless, radiopharmaceutical production, regulatory approval, acquisition standardisation, availability, and access to appropriate normative databases remain important practical determinants of PET implementation [10,23,24].

PET radiopharmaceuticals targeting AADC or VMAT2 should not be regarded simply as higher-resolution substitutes for DAT SPECT. [^18^F]FDOPA evaluates AADC-dependent dopamine synthesis and subsequent trapping, whereas tetrabenazine derivatives assess vesicular monoamine storage through VMAT2. Modality selection should therefore be based on the molecular process of interest, local expertise, radiopharmaceutical availability, and the purpose of imaging [4,14,16].

### 6.2. Presynaptic Versus Postsynaptic Dopaminergic Imaging

Presynaptic and postsynaptic imaging address different components of dopaminergic neurotransmission. Presynaptic radiopharmaceuticals establish whether nigrostriatal terminal function is impaired, whereas D_2_/D_3_ receptor imaging evaluates receptor availability and is additionally influenced by endogenous dopamine, receptor regulation, medication exposure, and disease stage [3,4,33,34,35].

Relatively preserved or compensatorily increased striatal D_2_/D_3_ receptor availability was historically considered supportive of PD, whereas reduced postsynaptic binding was proposed as a feature of atypical degenerative parkinsonism. In practice, substantial overlap exists among disorders, and receptor availability may change with disease progression and dopaminergic treatment. Postsynaptic imaging therefore provides limited incremental value for routine individual diagnosis and is used predominantly in research or selected specialised-centre evaluations [3,4,34,35].

Multitracer PET protocols combining DAT, AADC, VMAT2, or receptor imaging have contributed substantially to understanding compensatory mechanisms and the differential regulation of dopaminergic targets. However, they increase radiation exposure, cost, logistical complexity, and interpretative burden and should not be used routinely when a single presynaptic examination already answers the clinically relevant question [4,14,16].

### 6.3. Cerebral [^18^F]FDG PET

Unlike dopaminergic radiopharmaceuticals, [^18^F]fluorodeoxyglucose ([^18^F]FDG) does not directly assess nigrostriatal neurotransmission. It evaluates regional cerebral glucose metabolism and may identify disease-associated patterns involving cortical, subcortical, brainstem, and cerebellar networks. Its principal potential contribution arises when presynaptic dopaminergic dysfunction has already been demonstrated, but the distinction between PD and an atypical parkinsonian syndrome remains clinically unresolved [51,52,53].

PD is commonly associated with relatively preserved cortical metabolism during earlier motor stages, although network-based analyses may demonstrate a reproducible pattern of relative metabolic increases and decreases. MSA may show reduced metabolism in the putamen, pons, middle cerebellar peduncles, and cerebellum, with the distribution depending partly on the predominant clinical phenotype. PSP is more frequently associated with hypometabolism involving the medial frontal cortex, anterior cingulate region, caudate nuclei, thalami, and midbrain. CBS typically demonstrates asymmetric cortical and subcortical hypometabolism, often involving the frontoparietal and perirolandic regions contralateral to the more clinically affected side [51,52,53].

These metabolic patterns are probabilistic rather than pathognomonic. Their expression varies with disease stage, phenotype, cognitive involvement, image-processing method, and reader experience. Moreover, CBS is a clinical syndrome that may result from several underlying neuropathologies; an asymmetric metabolic pattern may support the clinical syndrome but cannot establish corticobasal degeneration as the pathological substrate. Visual interpretation may be strengthened by voxel-based or network analyses, although computational methods and their validation are considered separately in Section 7 [51,52,53].

Accordingly, [^18^F]FDG PET is most useful when the clinical question concerns the distribution or probable aetiology of a neurodegenerative process rather than the simple presence of presynaptic dopaminergic dysfunction. A normal or non-specific metabolic examination does not exclude an early atypical syndrome, whereas a characteristic disease-associated pattern should always be interpreted together with clinical findings and structural imaging [51,52,53].

### 6.4. Structural Magnetic Resonance Imaging

Structural MRI is complementary to molecular imaging and should form part of the assessment of clinically uncertain or atypical parkinsonism. Its first role is to identify alternative or contributing causes, including vascular lesions, normal-pressure hydrocephalus, tumour, demyelination, previous trauma, and other structural abnormalities capable of producing or modifying parkinsonian symptoms [2,8].

MRI may also demonstrate patterns that support an atypical neurodegenerative syndrome. MSA can be associated with putaminal atrophy and signal abnormalities, pontocerebellar atrophy, middle cerebellar peduncle involvement, and the pontine hot-cross-bun appearance. Midbrain and superior cerebellar peduncle atrophy may support PSP, whereas asymmetric frontoparietal or perirolandic atrophy may be observed in CBS. The current Movement Disorder Society diagnostic criteria for MSA incorporate specified MRI markers into the category of clinically established disease [54].

Quantitative MRI measurements may strengthen differential diagnosis at the group level. Midbrain area, the midbrain-to-pons ratio, and the magnetic resonance parkinsonism index are particularly informative for PSP, especially Richardson syndrome, whereas pontine measurements may support the diagnosis of MSA with predominant parkinsonism. However, diagnostic thresholds and performance remain incompletely harmonised across centres, and these indices should be regarded as supportive rather than definitive biomarkers [54,55]. Diffusion and other advanced quantitative MRI techniques may provide additional information, but routine cross-centre implementation remains limited by protocol variability and incomplete external validation [2,22,55].

Structural and dopaminergic imaging answer different questions. MRI may suggest a particular anatomical pattern or reveal a secondary cause, whereas DAT imaging establishes whether a significant presynaptic nigrostriatal deficit is present. Concordant abnormalities may increase diagnostic confidence, but discordance should prompt reconsideration of disease stage, phenotype, structural confounders, technical quality, and the original clinical diagnosis rather than automatic prioritisation of one modality [2,3,8,54].

### 6.5. Cardiac [^123^I]MIBG Scintigraphy

Cardiac scintigraphy with [^123^I]metaiodobenzylguanidine ([^123^I]MIBG) evaluates postganglionic sympathetic myocardial innervation rather than central dopaminergic function. Cardiac uptake is frequently reduced in Lewy body disorders, including PD and DLB, whereas it is more often preserved in atypical neurodegenerative parkinsonism, particularly MSA. It may therefore provide complementary information when the unresolved question concerns a Lewy body disease mechanism rather than the presence of nigrostriatal degeneration itself [45,56,57].

This distinction is not absolute. Preserved cardiac uptake may occur in early PD, whereas reduced uptake may occasionally be observed in patients with atypical parkinsonism. Cardiac disease, diabetes mellitus, peripheral autonomic neuropathy, medications interfering with noradrenaline transport or vesicular storage, and methodological differences in acquisition and heart-to-mediastinum quantification may produce false-positive or false-negative findings. Cardiac [^123^I]MIBG scintigraphy should therefore be interpreted as a supportive biomarker rather than an isolated pathological diagnosis [45,56,57].

In a retrospective head-to-head study of clinically challenging cases, [^18^F]FDG PET showed numerically higher diagnostic accuracy than cardiac [^123^I]MIBG scintigraphy for differentiating Lewy body from non-Lewy body disorders and PD from MSA. However, the differences in the consensus analyses did not reach statistical significance, and performance was influenced by reader experience. The two techniques should therefore be regarded as complementary: [^18^F]FDG PET evaluates cerebral neurodegenerative network patterns and may support classification of atypical parkinsonian syndromes, whereas [^123^I]MIBG assesses peripheral postganglionic sympathetic denervation [57].

### 6.6. Genetic Parkinsonism and Genotype-Associated Dopaminergic Imaging

Genetic background may influence the distribution and temporal evolution of presynaptic dopaminergic imaging abnormalities. In manifest Parkinson’s disease associated with pathogenic leucine-rich repeat kinase 2 (LRRK2) variants or glucosylceramidase beta 1 (GBA1) variants, striatal DAT loss is generally detectable, but comparisons with sporadic PD are heterogeneous and demonstrate substantial overlap rather than a diagnostic genotype-specific pattern [58]. Findings are even more variable among non-manifesting carriers. In a large multicentre cohort, a prespecified DAT deficit was present in 11% of non-manifesting LRRK2 carriers and 3% of non-manifesting GBA1 carriers with available imaging, while mean striatal DAT binding was higher in the GBA1 group than in healthy controls [59]. Across other GBA1 cohorts, preserved or increased DAT measurements coexist with isolated reports of mild DAT or VMAT2 reductions [58]. Small multitracer PET studies of non-manifesting LRRK2 carriers have also reported reduced DAT binding and, in some individuals, reduced VMAT2 binding despite preserved [^18^F]FDOPA uptake [58,60].

Parkinsonism associated with biallelic parkin RBR E3 ubiquitin protein ligase (PRKN) or PTEN-induced kinase 1 (PINK1) variants more often demonstrates bilateral or relatively symmetric striatal dopaminergic loss and may show slower longitudinal decline than typical late-onset PD [58,61,62]. However, these observations derive from small, heterogeneous cohorts examined with different radiopharmaceuticals, and human VMAT2 data remain particularly limited for PRKN- and PINK1-associated disease. Consequently, currently reported imaging patterns are informative at the group and mechanistic levels but cannot determine genotype in an individual patient; dopaminergic imaging should complement rather than replace molecular genetic testing [58].

### 6.7. Integrated Multimodal Diagnostic Strategies

A rational multimodal strategy begins with a clearly defined clinical question and structural brain imaging. When uncertainty concerns whether presynaptic nigrostriatal degeneration is present, DAT SPECT or an appropriate presynaptic PET examination is the most direct molecular imaging approach. When presynaptic imaging is abnormal, but the clinical phenotype contains red flags for MSA, PSP, or CBS, structural MRI and [^18^F]FDG PET may provide more useful aetiological information than repeating another examination directed at the same presynaptic target [2,3,8,51,54].

Cardiac [^123^I]MIBG scintigraphy may be considered when the distinction between Lewy body disease and a disorder expected to preserve postganglionic cardiac sympathetic innervation remains clinically important. Postsynaptic receptor imaging and multitracer dopaminergic PET should generally be reserved for specialised indications and research, because their incremental routine diagnostic value is limited and their signals are influenced by multiple biological and pharmacological variables [3,4,45,56,57].

Combined presynaptic and metabolic PET strategies have been evaluated in retrospective observational studies. Dual [^18^F]FDOPA–[^18^F]FDG PET/CT provides information on both presynaptic dopaminergic function and cerebral metabolic patterns and may influence diagnostic orientation in selected clinically complex patients. Nevertheless, the available studies included heterogeneous populations and relied predominantly on clinical rather than neuropathological reference diagnoses. Current evidence is therefore insufficient to demonstrate that routine dual-tracer imaging is superior to a sequential, question-driven strategy, particularly when radiation exposure, cost, availability, and the limited proportion of patients with long-term follow-up are considered [63,64].

The objective of multimodal imaging is not to accumulate concordant abnormalities, but to combine techniques that provide genuinely complementary information. Tests directed at the same molecular component may confirm the presence of a deficit without resolving its cause, whereas integration of presynaptic imaging, structural MRI, cerebral metabolism, and selected autonomic biomarkers can progressively narrow the differential diagnosis. The final interpretation should remain anchored in the clinical phenotype, longitudinal evolution, and the specific decision that imaging is intended to support [2,3,4,51,56,57].

Because these modalities interrogate different levels of disease biology, their selection should be guided by the unresolved clinical question rather than by their availability alone, as summarised in Table 2.

## 7. Quantification, Artificial Intelligence, and Emerging Approaches

Quantitative and computational methods extend visual assessment by improving reproducibility and extracting regional, spatial, or longitudinal information from borderline and heterogeneous examinations. Their outputs remain adjunctive to expert interpretation because they inherit the biological limitations of the molecular target and the technical dependencies of acquisition, reconstruction, processing, and reference data. In particular, neither numerical indices nor AI-derived outputs convert DAT availability into a direct neuronal count or overcome the aetiological overlap among degenerative parkinsonian syndromes [3,15,22,65,66,67].

### 7.1. Semiquantitative Analysis of DAT SPECT

Semiquantitative analysis of [^123^I]FP-CIT SPECT most commonly reports a specific binding ratio (SBR), generally calculated as (C_target_ − C_reference_)/C_reference_, where C denotes regional count concentration. Regions of interest (ROIs) or volumes of interest (VOIs) may encompass the caudate nucleus, putamen, or whole striatum, while the occipital cortex or another validated extrastriatal region is used to estimate nonspecific binding. Additional measures include left–right asymmetry, caudate-to-putamen ratio or its reciprocal, regional percentiles, and z-scores relative to normative data. Because anatomical VOI-based and whole-striatum count-based methods handle spill-out and partial-volume effects differently, values generated by different approaches should not be assumed to be equivalent [3,65,66,67].

Semiquantitative information can improve reporting reproducibility and reader confidence, especially in borderline examinations. In the study by Söderlund et al., adding SBR and caudate-to-putamen ratio data to visual assessment progressively improved interobserver agreement and reduced the number of examinations considered difficult. Quantification is therefore most useful when interpreted together with striatal morphology, clinical laterality, structural imaging, and image quality rather than applied as an isolated threshold-based decision. A convincing visual abnormality should not be rejected solely because a value lies within a reference interval, while an isolated abnormal numerical result should prompt review of processing, registration, structural abnormalities, and the selected reference database [3,65].

Normative comparison requires compatibility between the patient examination and the reference dataset. Relevant factors include camera and collimator characteristics, reconstructed spatial resolution, attenuation and scatter correction, filtering, spatial normalisation, ROI or VOI definition, reference-region selection, and the approach to partial-volume compensation. Reference values should also account for the age-related decline in DAT availability and, where appropriate, sex-related differences [3,66,67].

Consequently, SBR is a method-dependent index rather than an absolute measurement of transporter density, terminal number, neuronal survival, or disease severity. Values generated by different software packages or processing pipelines are not necessarily interchangeable, and longitudinal comparison is most reliable when acquisition and reconstruction remain stable. Phantom-based calibration and other harmonisation procedures can reduce inter-camera variability, but they cannot fully compensate for differences in reconstruction, subject-dependent effects, or a reference database that is incompatible with the local protocol [3,66,67].

### 7.2. Quantitative PET and Kinetic Modelling

Dynamic PET enables tracer-specific kinetic modelling. For reversible DAT radioligands such as [^18^F]FE-PE2I, reference-tissue models can estimate non-displaceable binding potential (BP_ND_), which represents specifically bound radioligand relative to the non-displaceable tissue compartment. Short static acquisitions can instead provide a standardised uptake value ratio (SUVR) between target and reference regions; when the same convention is used, SBR corresponds to SUVR − 1. The relationship of these ratio-based measures to BP_ND_ depends on the acquisition window, reference region, tracer clearance, and proximity to peak or pseudo-equilibrium [68,69].

Studies of [^18^F]FE-PE2I have shown that shortened static measurements can retain strong discrimination between patients with early PD and healthy controls when validated time windows are used. Delva et al. found that an early 15–40 min window produced the lowest SUVR bias relative to BP_ND_ + 1, whereas a 50–60 min window at pseudo-equilibrium offered clinically practical acquisition with high discriminative power. Brumberg et al. likewise found high discrimination for both early-peak and late pseudo-equilibrium ratios, but better test–retest reliability for the early window. These results support simplified protocols, but also show that values obtained from different windows should not be treated as interchangeable [68,69].

Test–retest performance is region-dependent. In patients with non-advanced PD, [^18^F]FE-PE2I BP_ND_ showed absolute variability of approximately 5.3–7.6% in striatal regions and 11% in the substantia nigra. The lower reliability of nigral measurements is consistent with the smaller anatomical volume and lower DAT density of this region, which increase susceptibility to partial-volume and segmentation effects [70].

Longitudinal [^18^F]FE-PE2I PET has detected progressive striatal DAT reductions in non-advanced PD. Among 25 patients with evaluable baseline and follow-up examinations separated by approximately 2.3 years, annualised decreases were 7.1–8.5% across the caudate nucleus, putamen, and sensorimotor striatum, whereas no clear longitudinal change was detected in the substantia nigra. These findings support the use of [^18^F]FE-PE2I as a research progression marker, but repeatability and measurable decline do not by themselves establish a validated surrogate endpoint for clinical progression or treatment efficacy. Target regulation, disease stage, treatment exposure, measurement variability, and the non-linear relationship between DAT availability and clinical impairment remain important constraints [3,15,22,71].

Quantitative outcomes are also radiopharmaceutical-specific. For [^18^F]FDOPA, dynamic analysis commonly estimates an influx constant (K_i_) related to AADC-dependent processing and subsequent tracer retention rather than DAT binding. VMAT2 and receptor radioligands require their own kinetic models and outcome measures. BP_ND_, SUVR, SBR, and K_i_ therefore represent different biological and mathematical quantities and should be interpreted using tracer- and model-specific frameworks [4,14,16,28,68,69,70,71].

### 7.3. AI-Assisted Image Interpretation

AI approaches applied to dopaminergic imaging range from machine learning (ML) models based on predefined quantitative and demographic features to convolutional neural networks (CNNs) that analyse reconstructed images directly. In the available DAT SPECT literature, the principal applications include binary classification of normal versus reduced uptake, categorical analysis of caudate and putaminal subregions, and identification of examinations for which the automated classification is uncertain [72,73,74].

In a two-centre study, a support vector machine using striatal uptake ratios, age, and sex achieved 95% accuracy in unseen data from the development centre, identical to the physicians’ accuracy, and 82.5% accuracy in the external centre, where performance remained comparable to expert interpretation. The numerical decline in external performance illustrates both the potential value of feature-based decision support and the need for independent external testing across centres with different, but sufficiently compatible, acquisition and processing protocols [72].

Deep-learning models can analyse the spatial uptake pattern without requiring manually predefined striatal indices. A recent CNN developed for scanner-independent five-level categorisation achieved regional accuracies of approximately 76.5–80.1% in caudate and putaminal subregions across independent and out-of-distribution test datasets. However, all four regional categories were simultaneously correct in only approximately 53% of examinations. Thus, detailed multi-level regional classification remains more difficult than global normal-versus-reduced categorisation, particularly for borderline patterns and image characteristics that are insufficiently represented during training [73,74].

A clinically useful system should communicate uncertainty rather than force every examination into a binary category. In the study by Budenkotte et al., an uncertainty-detection module flagged only 4.3% of examinations in the development test set but identified 90% of the misclassified cases; similar behaviour was observed in internal and external test datasets. Such systems could direct expert attention towards borderline or technically atypical examinations, but their proposed clinical benefit still requires prospective confirmation, and final interpretation must remain with the physician [73].

The reference standard used for AI development is a major limitation. Labels may be derived from visual interpretation, semiquantitative thresholds, clinical diagnosis, or combinations of these sources. A model may therefore reproduce reader disagreement, database-specific thresholds, or diagnostic misclassification present in the training data. High agreement with an expert read demonstrates reproduction of an imaging classification task; it does not prove PD, establish neuropathology, or reliably distinguish individual degenerative parkinsonian syndromes [2,3,72,73,74].

Overall, the cited AI evidence is predominantly retrospective or based on secondary analyses of existing datasets, with recurrent but not exclusive use of the Parkinson’s Progression Markers Initiative dataset; generalisability therefore remains uncertain, and future studies should follow contemporary AI reporting guidance [72,73,74,75,76,77].

### 7.4. Radiomics and Predictive Modelling

Radiomics and engineered-feature analysis seek to characterise spatial information that may not be captured by mean regional SBR alone. Potential descriptors include uptake heterogeneity, texture, asymmetry, regional gradients, shape, and relationships among functional striatal subdivisions. These features can be combined with demographic and clinical variables in statistical or ML models intended to estimate diagnosis or subsequent clinical outcome [75].

In a secondary analysis of 69 participants from the Parkinson’s Progression Markers Initiative, Tang et al. combined baseline DAT SPECT radiomic features with clinical information to predict motor outcome four years later. The study demonstrated the feasibility of extracting prognostic information beyond binary scan classification, but the limited cohort and absence of independent external testing preclude its use for individual prognosis, patient counselling, or treatment selection [75].

Predictive models are particularly vulnerable to overfitting when the number of imaging features is large relative to the cohort size. Feature selection, model tuning, and performance assessment should therefore be separated appropriately, preferably through nested validation followed by independent external testing. Multimodal models should also demonstrate incremental value beyond established clinical predictors rather than merely reconstructing information already available to the treating neurologist [72,75,76].

### 7.5. Emerging Acquisition and Multiparametric Approaches

Advances in detector and collimator design may improve DAT SPECT efficiency. In a single-centre retrospective series of 640 examinations acquired with a triple-head system and second-generation multiple-pinhole brain collimators, image quality remained diagnostic for all patients at a simulated 12 min acquisition, and disagreement between 30 and 12 min visual interpretations was comparable to intra-reader variability. These results support shorter acquisition on the evaluated system. Still, they should not be extrapolated directly to conventional parallel-hole systems because performance depends on camera geometry, collimator design, reconstruction, administered activity, and validation procedure [78].

Dynamic PET may yield more than one parameter from a single radiopharmaceutical administration. For [^18^F]FE-PE2I, the relative tracer-delivery parameter R_1_ derived by reference-tissue modelling has been evaluated as a proxy for relative regional cerebral blood flow, while BP_ND_ provides DAT availability from the same dynamic dataset. Comparison with [^15^O]H_2_O PET demonstrated strong regional correlations and good-to-excellent agreement, supporting the feasibility of combining perfusion-related and presynaptic dopaminergic information in one examination [79].

R_1_ derived from [^18^F]FE-PE2I should not, however, be considered equivalent to cerebral glucose metabolism measured with [^18^F]FDG PET or assumed to replace an independently validated perfusion study in every clinical setting. Its acquisition requirements, regional reliability, and incremental diagnostic value require further validation. Similarly, faster SPECT, simplified PET protocols, and multiparametric acquisitions should be regarded as workflow and information-efficiency innovations rather than intrinsically superior diagnostic tests [22,51,78,79].

### 7.6. Requirements for Clinical Translation

Clinical implementation of quantitative and AI-assisted approaches requires more than high standalone classification accuracy. Essential elements include a clearly defined intended use, representative multicentre data, compatibility or harmonisation across scanners and reconstruction protocols, independent external testing, calibrated outputs, explicit management of uncertain and out-of-distribution examinations, and transparent documentation of model inputs and reference standards [66,67,72,73,74,76].

Prospective studies should determine whether these tools improve reader agreement, reduce reporting time, decrease clinically relevant errors, or meaningfully influence patient management. Automated results should be presented in a form that permits verification of regional measurements and recognition of technical failure. Systems that provide interpretable regional outputs and indicate when expert review is required are more clinically credible than algorithms that generate an unsupported disease label [65,72,73,74,76].

Quantification and AI are therefore most appropriately positioned as decision-support tools. Semiquantitative indices can contextualise borderline DAT availability, kinetic PET can provide tracer-specific measurements, and computational models can integrate complex spatial information or flag uncertain cases. Their ultimate value will depend on reproducibility, prospective clinical utility, and their ability to improve decisions rather than simply maximise classification performance.

Table 3 provides a comparative overview of the principal outputs, potential advantages, limitations, and current implementation status of these quantitative, computational, and emerging approaches.

## 8. Future Perspectives

The use of dopaminergic imaging as a progression biomarker or surrogate endpoint presents a separate and more demanding challenge. A biomarker may distinguish patients from healthy controls and demonstrate longitudinal change without reliably reflecting clinically meaningful progression or therapeutic benefit. DAT availability may be affected by compensatory regulation, pharmacological exposure, disease stage, and floor effects in severely affected striatal regions, whereas clinical deterioration also reflects pathology outside the nigrostriatal system. Future longitudinal studies should therefore establish tracer- and region-specific measurement error, minimal detectable change, trajectories across disease stages, and associations between imaging changes and patient-centred outcomes. Importantly, reduced striatal DAT binding assessed by SPECT has been qualified for a defined trial-enrichment context in early motor PD, but this should not be conflated with validation as a monitoring biomarker or surrogate endpoint for treatment efficacy [4,14,17,22,71,80].

Technical advances may improve measurement precision and workflow efficiency, but their translational value ultimately depends on harmonised validation and demonstrable clinical utility; none can overcome the limited disease specificity of the underlying biological target. The same principle applies to computational tools, for which prospective workflow-based utility is more informative than incremental improvements in retrospective classification accuracy [3,66,67,72,73,74,75,76,78,79].

### 8.1. DAT Imaging in Prodromal Synucleinopathy

The clinical significance of a normal or borderline examination depends on whether imaging is performed after motor parkinsonism has emerged or during a prodromal state. In clinically manifest early motor PD, presynaptic DAT loss is generally detectable [3,18]. In prodromal populations, however, sensitivity is inherently time-dependent because the reference outcome is subsequent phenoconversion rather than a contemporaneous clinical diagnosis. Reduced DAT binding in individuals with polysomnography-confirmed isolated rapid eye movement sleep behaviour disorder (iRBD) or hyposmia identifies subgroups at increased short-term risk. In one longitudinal cohort of iRBD, baseline DAT SPECT predicted phenoconversion to a clinically defined synucleinopathy with 75% sensitivity and 51% specificity at five years [81]. In the Parkinson Associated Risk Study, 67% of hyposmic participants with a baseline DAT deficit developed PD within four years; however, conversion also occurred in 9% of those with indeterminate binding and 2.8% of those without a baseline DAT deficit [82]. Thus, normal or indeterminate dopaminergic imaging cannot exclude the later development of PD or another synucleinopathy in a prodromal individual. Accordingly, the Movement Disorder Society exclusion criterion concerning normal presynaptic dopaminergic imaging should not be extrapolated to individuals who have not yet developed parkinsonism [1].

More recent multicentre studies reinforce the potential value of DAT imaging for risk stratification in iRBD. In an international cohort of 263 individuals with iRBD, 52 (20%) developed a clinically defined synucleinopathy during an average follow-up of two years, and the strongest combination of short-term risk factors comprised reduced putaminal DAT binding, constipation, and age older than 70 years [83]. In a subsequent retrospective multicentre analysis, a machine-learning model combining clinical and presynaptic dopaminergic imaging variables distinguished individuals who subsequently phenoconverted from those who had not phenoconverted by the last follow-up with 77% sensitivity and 85% specificity [84]. An expanded international multicentre study published in 2026 included 1,067 participants, of whom 400 had iRBD, and found that proposed putaminal z-score strata predicted phenoconversion, with hazard ratios ranging from 3.10 to 5.03, while outperforming selected clinical risk measures [85]. These findings support DAT imaging primarily for research-cohort enrichment, neurodegeneration staging, and trial stratification. However, cohort-derived thresholds and the absence of independent prospective validation currently limit their routine application for prediction at the individual-patient level [83,84,85].

### 8.2. α-Synuclein PET: Towards Pathology-Specific Imaging

A major future direction in molecular imaging of parkinsonian disorders is the transition from visualising downstream neurotransmitter dysfunction towards identifying the defining molecular pathology. PET radiopharmaceuticals targeting aggregated α-synuclein could potentially provide an in vivo marker of the pathological process underlying PD, DLB, and MSA. This would represent a conceptually different approach from DAT imaging, which assesses presynaptic nigrostriatal dysfunction through transporter availability but does not directly identify the underlying proteinopathy. Nevertheless, as of June 2026, no α-synuclein PET radiopharmaceutical was approved for routine clinical use, and all available human studies should be considered preliminary proof-of-concept investigations [86,87,88].

Among the investigational tracers evaluated in humans, [^18^F]ACI-12589 has undergone the most extensive clinical characterisation. Its initial clinical evaluation included 42 participants: 8 healthy controls (HCs), 8 patients with PD, 2 with DLB, 13 with MSA, and 11 with other neurodegenerative disorders. Patients with MSA demonstrated increased tracer retention predominantly in the cerebellar white matter and middle cerebellar peduncles, with more extensive involvement in MSA with predominant cerebellar ataxia (MSA-C). By contrast, clinically meaningful retention was not consistently demonstrated in idiopathic PD or DLB. Although the initial characterisation of ACI-12589 indicated favourable in vitro selectivity over amyloid-β (Aβ) and tau aggregates, subsequent independent post-mortem autoradiography using tritiated ACI-12589 reported evidence of off-target binding to Aβ plaques. Together with its α-synuclein affinity in the tens-of-nanomolar range, this finding supports the need for further selectivity validation, particularly in neuronal Lewy-type synucleinopathies, where the pathological α-synuclein burden is lower than in MSA [86,88,89].

[^18^F]C05-05 has produced a different and potentially complementary imaging pattern. Increased midbrain retention was reported in small cohorts of patients with PD and DLB, whereas increased uptake in the putamen and middle cerebellar peduncles was observed in patients with MSA. These findings suggest that [^18^F]C05-05 may provide disease-associated regional binding patterns across both neuronal and oligodendroglial synucleinopathies, although its pathological specificity remains to be established. However, the available cohorts remain small, considerable overlap exists between patients and HCs, and the tracer exhibits relatively high background activity. Moreover, although the ligand was developed to favour α-synuclein binding, comparative in vitro data have raised concerns regarding potential residual binding to Aβ and tau assemblies. The extent to which such cross-reactivity could affect clinical interpretation in patients with mixed proteinopathies remains uncertain [87,88].

Initial human imaging with [^18^F]SPAL-T-06 included only three patients with MSA—two with MSA with predominant parkinsonism (MSA-P) and one with MSA-C—and one HC. Increased retention was observed in the putamen, pons, cerebellar white matter, and cerebellar peduncles, broadly corresponding to the expected distribution of α-synuclein-containing glial cytoplasmic inclusions in MSA. Rapid cerebral clearance produced comparatively low background activity, representing a potentially favourable imaging characteristic. However, the extremely small cohort, the absence of a formal semiquantitative group comparison, the limited published preclinical characterisation, and the lack of comparable data in PD and DLB preclude conclusions regarding diagnostic performance [90,91].

The carbon-11-labelled tracer [^11^C]MODAG-005 has demonstrated subnanomolar binding affinity for recombinant α-synuclein fibrils and α-synuclein inclusions in human brain tissue, together with favourable brain penetration and clearance in experimental models. Preclinical studies also demonstrated target engagement by the aggregation inhibitor anle138b, suggesting a potential role in therapeutic development studies. However, first-in-human [^11^C]MODAG-005 PET imaging was performed in only four participants: one patient with MSA-C, one with combined cerebellar and parkinsonian MSA features, one with GBA1-associated PD, and one asymptomatic participant considered an HC by the investigators, without a formal group-level evaluation of diagnostic performance. Consequently, the observed regional retention patterns cannot yet be translated into diagnostic thresholds or estimates of sensitivity and specificity. Furthermore, the short physical half-life of carbon-11 restricts its use to specialised centres with an on-site cyclotron and appropriate radiochemistry infrastructure [92].

By contrast, the first-in-human evaluation of [^11^C]HY-2-15 provided less favourable translational results. The pilot study included ten participants comprising HCs and patients with PD, MSA, or PSP. No significant disease-related differences in uptake were demonstrated in PD or MSA. Although relatively increased retention was observed in the pallidum and midbrain of patients with PSP, this finding did not reach statistical significance and was consistent with the tracer’s additional affinity for tau pathology. Rapid metabolism, low initial brain uptake, and limited specific cerebral signal further restricted its imaging performance. These findings do not support [^11^C]HY-2-15 as a selective α-synuclein imaging agent in its current form, but they provide valuable information regarding the physicochemical and pharmacokinetic requirements for future tracer development [93].

Human biodistribution and radiation dosimetry have also been reported for [^18^F]SPAL-T-06 and [^18^F]C05-05 in two healthy participants per tracer. Both radiopharmaceuticals were well tolerated in these small cohorts. Following an administered activity of 185 MBq, the estimated effective doses were 6.6 mSv for [^18^F]SPAL-T-06 and 5.6 mSv for [^18^F]C05-05, values comparable to those of other fluorine-18-labelled PET radiopharmaceuticals used for imaging neurodegenerative proteinopathies [91]. The principal investigational α-synuclein PET tracers for which peer-reviewed human imaging data were available by June 2026 are critically compared in Table 4.

Several biological and technical factors make α-synuclein a particularly challenging molecular imaging target. Compared with Aβ plaques, pathological α-synuclein aggregates occur at substantially lower concentrations and are predominantly intracellular. An effective radiotracer must therefore cross both the BBB and cellular membranes while maintaining sufficiently high affinity, selectivity, and reversible binding kinetics. High lipophilicity may facilitate brain entry but can simultaneously increase non-specific binding, particularly in cerebral white matter, thereby reducing the target-to-background ratio [86,87,88].

α-Synuclein aggregates are also structurally and biologically heterogeneous. Neuronal Lewy bodies and Lewy neurites in PD and DLB differ from the predominantly oligodendroglial cytoplasmic inclusions encountered in MSA with respect to their cellular localisation, abundance, fibrillar organisation, and conformational strain. A radiotracer optimised for MSA-associated aggregates may therefore not demonstrate equivalent binding to Lewy-type pathology in PD or DLB. Furthermore, binding characteristics established using recombinant preformed fibrils may not accurately reproduce interactions with native patient-derived aggregates, potentially explaining discrepancies between promising preclinical results and limited human imaging performance [86,87,88,90,92,93].

Additional challenges include potential cross-reactivity with Aβ or tau aggregates, binding to other molecular targets such as monoamine oxidase B, non-specific retention in cerebral grey or white matter, suboptimal brain penetration, rapid peripheral metabolism, and the formation of brain-penetrant radiometabolites. Quantification is further complicated by partial-volume effects, small anatomically relevant target regions, disease-dependent regional involvement, and the absence of a universally valid reference region devoid of specific binding. Finally, the lack of an ante-mortem diagnostic gold standard limits definitive validation. Longitudinal studies incorporating standardised kinetic modelling, independent biomarker confirmation, and eventual scan-to-autopsy correlation will therefore be essential [86,87,88,90,91,92,93].

If these limitations can be overcome, α-synuclein PET could introduce a pathology-specific layer into molecular imaging algorithms for parkinsonian disorders. Current dopaminergic imaging primarily addresses whether presynaptic nigrostriatal dysfunction is present and characterises its anatomical distribution. It cannot reliably determine whether the underlying process represents a neuronal synucleinopathy, an oligodendroglial synucleinopathy, a tauopathy, or another neurodegenerative disorder. In a future diagnostic framework, dopaminergic imaging could continue to demonstrate functional impairment of the nigrostriatal system, whereas α-synuclein PET could potentially identify the molecular substrate responsible for that impairment [2,3,4,22,86,87,88].

Such an approach might facilitate differentiation between synucleinopathies and non-synucleinopathic parkinsonian disorders and, if reproducible disease-specific patterns are established, between oligodendroglial MSA pathology and neuronal Lewy-type pathology in PD or DLB. However, a negative α-synuclein PET examination cannot currently exclude a synucleinopathy, particularly PD or DLB, because the available tracers have not demonstrated adequate and independently replicated sensitivity for neuronal Lewy-type pathology. Similarly, the ability to distinguish PD from DLB or to separate individual atypical parkinsonian syndromes has not yet been established [86,87,88,90,92,93].

The most immediate applications of α-synuclein PET may therefore lie in patient selection for disease-modifying clinical trials, confirmation of therapeutic target engagement, and longitudinal assessment of interventions directed against α-synuclein aggregation. Before clinical implementation, future studies must demonstrate reproducibility, validated quantitative thresholds, diagnostic accuracy in clinically relevant populations, incremental value over neurological assessment and established biomarkers, and a measurable effect on patient management. At present, α-synuclein PET should consequently be regarded as complementary to, rather than a replacement for, established dopaminergic imaging [22,86,87,88,90,91,92,93].

Alongside PET, α-synuclein seed amplification assays (αSyn-SAAs) provide a pathology-directed approach by detecting seeding-competent misfolded α-synuclein in biological specimens. Cerebrospinal fluid (CSF) αSyn-SAA has shown high diagnostic performance in a large multicentre PD cohort, although sensitivity varied across phenotypic and genetic subgroups [94]. Skin-based αSyn-SAA has shown promising proof-of-concept and pooled diagnostic performance, but methodological heterogeneity and the need for further standardisation currently limit routine interpretation [95,96]. The 2024 neuronal α-synuclein disease integrated staging system (NSD-ISS) and SynNeurGe research frameworks formally incorporate pathology and neurodegeneration biomarkers: the NSD-ISS uses pathological neuronal α-synuclein (S) and dopaminergic neuronal dysfunction (D) as distinct biological anchors, whereas SynNeurGe integrates α-synuclein pathology (Syn), neurodegeneration defined by neuroimaging (Neur), and pathogenic genetic variants (Ge) [97,98]. Within these frameworks, abnormal DAT imaging can provide evidence for D+ status in the NSD-ISS or N+ status in SynNeurGe, rather than serving as a standalone disease label. Consequently, after a conclusive positive αSyn-SAA result, the incremental value of DAT imaging depends on whether confirmation or anatomical characterisation of nigrostriatal dysfunction would influence clinical interpretation or management. Both frameworks are currently intended for research use and do not replace established clinical diagnostic criteria [97,98].

### 8.3. Pathology-Informed Multimodal Strategies

Beyond α-synuclein PET, future multimodal assessment should be structured around the biological level that remains unresolved after clinical evaluation and dopaminergic imaging. Metabolic or perfusion imaging, structural MRI, autonomic imaging, genetic testing, and fluid biomarkers should be added selectively when they provide non-redundant information regarding aetiology, disease distribution, or therapeutic eligibility. The value of any multimodal combination must be demonstrated through incremental diagnostic, prognostic, or predictive performance and, ultimately, through a measurable effect on patient management [2,3,4,22].

Future progress will therefore depend on aligning each biomarker with a precise clinical or therapeutic question. Pathology-specific radiopharmaceuticals, standardised longitudinal measurements, and prospectively validated multimodal models may extend the role of dopaminergic imaging, but only if the distinction between what an examination measures, what disease it suggests, and what decision it changes is preserved [2,3,4,22].

## 9. Conclusions

Dopaminergic radiopharmaceutical imaging provides an objective in vivo assessment of nigrostriatal function. In routine practice, [^123^I]FP-CIT SPECT remains the most established method for demonstrating or arguing against a presynaptic nigrostriatal dopaminergic deficit in clinically uncertain parkinsonian syndromes, whereas PET radiopharmaceuticals targeting DAT, AADC, or VMAT2 provide complementary target-specific information in selected settings. An abnormal examination should not be equated with PD, because presynaptic dopaminergic dysfunction is shared by several degenerative and acquired disorders and does not identify the underlying neuropathology.

On this basis, four practical recommendations follow. First, before imaging is requested, the clinical decision that would be changed by a normal, abnormal, or equivocal result should be defined. Second, reports should identify the molecular target, describe the presence and regional distribution of the abnormality, and state relevant interpretive limitations without assigning an unsupported aetiological label. Third, borderline or clinically discordant findings should prompt review of medication exposure, technical quality, semiquantitative results, structural imaging, and clinical evolution before repeat or additional imaging is considered. Fourth, complementary structural, metabolic, autonomic, genetic, or pathology-directed biomarkers should be added only when they address a non-redundant question; quantitative and AI-assisted outputs should support rather than replace expert interpretation, and emerging approaches require prospective multicentre validation before broader implementation.

## Figures and Tables

**Table 1:** Principal dopaminergic radiopharmaceuticals and their molecular imaging characteristics.

Molecular Target	Radiopharmaceuticals	Imaging Modality	Mechanism and Biological Information	References
DAT	[^123^I]FP-CIT	SPECT	Reversible binding to DAT on presynaptic dopaminergic terminals; striatal uptake reflects DAT availability and serves as a surrogate marker of presynaptic nigrostriatal terminal integrity.	[3,17,18,19]
DAT	[^123^I]β-CIT, [^123^I]IPT, [^123^I]altropane, [^123^I]PE2I, [^99m^Tc]TRODAT-1	SPECT	Tropane-derived radioligands that bind presynaptic DAT, with tracer-dependent differences in transporter selectivity, cerebral kinetics, and nonspecific binding.	[3,17,19,20,21]
DAT	[^18^F]FP-CIT, [^18^F]FE-PE2I	PET	Reversible binding to presynaptic DAT, enabling high-resolution assessment and quantification of striatal DAT availability. Under validated conditions, [^18^F]FE-PE2I may also assess DAT availability in selected midbrain regions.	[4,10,23,24]
DAT	[^11^C]PE2I, [^18^F]FECNT, [^18^F]LBT-999	PET	Selective or relatively selective reversible binding to DAT, with tracer-dependent differences in affinity, kinetics, metabolism, and regional distribution.	[5,14,16,22,25,26,27]
AADC	[^18^F]FDOPA	PET	Transported across the BBB through LAT1, decarboxylated by AADC to [^18^F]fluorodopamine, and subsequently sequestered into synaptic vesicles by VMAT2. The signal reflects AADC-dependent dopamine synthesis capacity and subsequent vesicular trapping.	[3,4,5,14,15,16,28]
AADC	[^18^F]FMT	PET	Decarboxylated by AADC to [^18^F]fluorometatyramine, followed predominantly by MAO-dependent metabolic trapping rather than substantial VMAT2-mediated vesicular storage.	[16,29,30]
VMAT2	(+)-[^11^C]DTBZ, [^18^F]AV-133	PET	Reversible binding to VMAT2 on monoaminergic synaptic vesicles; striatal binding reflects vesicular storage capacity and serves as a surrogate marker of predominantly nigrostriatal monoaminergic terminal integrity.	[4,14,15,16,31,32]
D_2_/D_3_ receptors	[^11^C]raclopride, [^18^F]fallypride, [^18^F]DMFP, [^123^I]IBZM, [^123^I]epidepride	PET/ SPECT	Reversible binding to D_2_/D_3_ receptors. Lower-affinity ligands predominantly assess striatal receptor availability, whereas higher-affinity ligands also permit evaluation of lower-density extrastriatal receptor populations. Binding is influenced by endogenous dopamine, receptor regulation, disease stage, and medication exposure.	[3,4,33,34,35,36]

Abbreviations: [^11^C] = carbon-11; [^18^F] = fluorine-18; [^99m^Tc] = technetium-99m; [^123^I] = iodine-123; AADC = aromatic L-amino acid decarboxylase; BBB = blood–brain barrier; β-CIT = 2β-carbomethoxy-3β-(4-iodophenyl)tropane; D_2_/D_3_ = dopamine D_2_/D_3_ receptor subtypes; DAT = dopamine transporter; DMFP = desmethoxyfallypride; DTBZ = dihydrotetrabenazine; FDOPA = 6-fluoro-L-DOPA; FMT = 6-fluoro-L-meta-tyrosine; FP-CIT = ioflupane; IBZM = iodobenzamide; LAT1 = large neutral amino acid transporter type 1; MAO = monoamine oxidase; PET = positron emission tomography; SPECT = single-photon emission computed tomography; VMAT2 = vesicular monoamine transporter type 2. IPT, PE2I, TRODAT-1, FE-PE2I, FECNT, LBT-999, and AV-133 are conventional radiotracer names or development codes.

**Table 2:** Question-driven comparative and complementary roles of imaging strategies in parkinsonian syndromes.

Unresolved Clinical Questions	Major Imaging Strategies	Main Contributions	Principal Limitations	References
Is clinically significant presynaptic nigrostriatal dysfunction present?	[^123^I]FP-CIT SPECT; DAT PET with [^18^F]FP-CIT or [^18^F]FE-PE2I, where available	Demonstrates or argues against a presynaptic DAT deficit. DAT PET may provide higher-resolution regional assessment and more flexible kinetic quantification.	An abnormal result does not establish aetiology or reliably distinguish PD from MSA, PSP, CBS, or DLB. PET availability and standardisation remain limited.	[2,3,4,8,10,23,24]
Is there a structural cause or an imaging pattern supporting an atypical degenerative syndrome?	Structural MRI and/or cerebral [^18^F]FDG PET	MRI identifies structural mimics and may demonstrate supportive anatomical markers; [^18^F]FDG PET characterises disease-associated metabolic patterns involving cortical, subcortical, brainstem, and cerebellar networks.	Findings are supportive rather than pathognomonic and may overlap across disorders, particularly during early disease. Performance depends on phenotype, disease stage, acquisition, and analytical method.	[2,8,51,52,53,54,55]
Does the phenotype raise a specific question of Lewy body disease versus MSA or another non-Lewy body disorder?	Cardiac [^123^I]MIBG scintigraphy, interpreted with clinical findings and cerebral imaging	Evaluates postganglionic cardiac sympathetic innervation and provides information complementary to striatal DAT availability.	Cardiac disease, diabetes, autonomic neuropathy, medication exposure, and protocol variability may affect uptake. Preserved uptake may occur in early PD, whereas reduced uptake is not entirely specific to Lewy body disease.	[45,56,57]
Is tracer-specific characterisation of another presynaptic process required?	[^18^F]FDOPA PET or VMAT2 PET	Evaluates AADC-dependent dopamine synthesis and trapping or vesicular monoamine storage, respectively, providing biological information complementary to DAT imaging.	These targets are differently regulated and do not provide interchangeable estimates of neuronal survival. Availability is limited, and their use remains predominantly research-oriented or restricted to specialised centres.	[4,14,16,31,32]
Is postsynaptic receptor status itself the specialised or research question?	D_2_/D_3_ receptor PET or SPECT	Assesses postsynaptic receptor availability and may contribute to the investigation of dopaminergic receptor regulation.	Considerable overlap among parkinsonian disorders, together with the effects of endogenous dopamine, medication, and disease stage, limits incremental value for routine individual diagnosis.	[3,4,33,34,35]
Would combined presynaptic and metabolic information help resolve a selected complex presentation?	Sequential [^18^F]FDOPA and [^18^F]FDG PET	Combines evidence of presynaptic dopaminergic dysfunction with cerebral metabolic patterns that may support aetiological classification.	Evidence is based predominantly on observational cohorts with heterogeneous populations and clinical reference diagnoses. The incremental benefit of routine dual-tracer imaging over selective, sequential imaging remains uncertain, while dual-tracer protocols increase radiation exposure, cost, and logistical complexity.	[51,63,64]

Abbreviations: [^18^F] = fluorine-18; [^123^I] = iodine-123; AADC = aromatic L-amino acid decarboxylase; CBS = corticobasal syndrome; D_2_/D_3_ = dopamine D_2_/D_3_ receptor subtypes; DAT = dopamine transporter; DLB = dementia with Lewy bodies; FDG = fluorodeoxyglucose; FDOPA = 6-fluoro-L-DOPA; FP-CIT = ioflupane; MIBG = metaiodobenzylguanidine; MRI = magnetic resonance imaging; MSA = multiple system atrophy; PD = Parkinson’s disease; PET = positron emission tomography; PSP = progressive supranuclear palsy; SPECT = single-photon emission computed tomography; VMAT2 = vesicular monoamine transporter type 2.

**Table 3:** Comparative overview of quantitative, computational, and emerging approaches in dopaminergic imaging.

Approach	Principal Outputs	Main Potential Advantages	Principal Limitations	Current Evidence and Implementation Status	References
Semiquantitative DAT SPECT	SBR, asymmetry indices, regional z-scores	Improves reproducibility and supports interpretation of borderline examinations	Dependence on acquisition, reconstruction, software, and normative database	Established clinical adjunct to visual interpretation	[3,65,66,67]
Dynamic DAT PET kinetic modelling	BP_ND_ and other model-derived parameters	Target-specific quantification and assessment of longitudinal change	Longer acquisition, motion sensitivity, and modelling requirements	Predominantly research and specialised-centre use	[3,68,70,71]
Simplified static DAT PET	SUVR or SBR, depending on the analytical convention	Shorter and more practical acquisition than full kinetic modelling	Dependence on acquisition window, reference region, and tracer kinetics	Clinical feasibility demonstrated; routine implementation remains limited	[68,69]
AI-assisted DAT SPECT classification	Binary or regional uptake category and uncertainty estimate	Automated categorisation, improved consistency, and prioritisation of uncertain examinations	Dataset shift, label dependence, limited explainability, and uncertain prospective clinical benefit	Investigational decision support; predominantly retrospective evidence	[72,73,74,76]
Radiomics and predictive modelling	Multivariate imaging signature or predicted clinical outcome	May extract prognostic information beyond mean regional uptake	High risk of overfitting and limited independent external testing	Exploratory research	[75,76]
Accelerated multiple-pinhole DAT SPECT	Short-duration reconstructed images and regional uptake measures	Shorter examination, improved patient tolerance, and potentially reduced motion	System-, collimator-, reconstruction-, and protocol-specific performance	Clinical feasibility demonstrated for selected dedicated systems	[78]
Multiparametric dynamic [^18^F]FE-PE2I PET	DAT BP_ND_ and relative tracer-delivery parameter R_1_	Combines presynaptic dopaminergic and perfusion-related information in one examination	Limited validation of regional reliability and incremental diagnostic value	Research application	[79]

Abbreviations: [^18^F] = fluorine-18; AI = artificial intelligence; BP_ND_ = non-displaceable binding potential; DAT = dopamine transporter; PET = positron emission tomography; R_1_ = relative tracer-delivery parameter; SBR = specific binding ratio; SPECT = single-photon emission computed tomography; SUVR = standardised uptake value ratio.

**Table 4:** Critical comparison of investigational α-synuclein PET radiotracers evaluated in humans.

Radiotracer	Predominant Human Imaging Findings	Principal Potential Advantage	Main Limitations and Current Status	References
[^18^F]ACI-12589	Increased retention in the cerebellar white matter and middle cerebellar peduncles in MSA, particularly in MSA-C, with limited retention in PD and DLB.	Disease-associated regional contrast demonstrated in MSA, particularly MSA-C.	Limited and variable retention in Lewy body-predominant disorders; small clinical cohorts; phenotype-specific diagnostic performance remains unestablished. Independent post-mortem autoradiography using tritiated ACI-12589 provided evidence of off-target binding to Aβ plaques, warranting further selectivity validation.	[86,88,89]
[^18^F]C05-05	Increased midbrain retention in PD and DLB and involvement of the putamen and middle cerebellar peduncles in MSA.	Potential visualisation of disease-associated regional binding patterns across different synucleinopathies.	Relatively high background activity, overlap between groups, and small clinical cohorts. Potential in vitro cross-reactivity with Aβ and tau may further limit specificity, although its clinical relevance remains uncertain.	[87,88]
[^18^F]SPAL-T-06	High-contrast retention in the putamen, pons, cerebellar white matter, and cerebellar peduncles was reported in patients with MSA.	Promising disease-associated contrast in MSA combined with the practical distribution advantages of fluorine-18 labelling.	Evaluated in only three patients with MSA and one HC, without formal group-level quantitative comparison; independent validation and more extensive selectivity data are required.	[88,90]
[^11^C]MODAG-005	Brain penetration and pharmacokinetic feasibility were demonstrated in the first human evaluation, but disease-specific binding has not yet been established.	Subnanomolar in vitro affinity and favourable preclinical brain kinetics support further clinical development.	Evaluated in only four participants (two with MSA, one with GBA1-associated PD, and one asymptomatic participant considered an HC) without formal group-level evaluation; disease-discriminating performance remains unknown, while carbon-11 labelling limits widespread clinical implementation.	[92]
[^11^C]HY-2-15	Human PET imaging was feasible, but no statistically significant discrimination between PD or MSA and HCs was demonstrated in the initial pilot study.	Human imaging feasibility was demonstrated, and the study provided initial kinetic and whole-body biodistribution data that may guide further ligand optimisation.	Low brain uptake, rapid metabolism, affinity for tau, and absence of significant group discrimination currently limit its potential as an α-synuclein-specific radiotracer.	[93]

Abbreviations: [^11^C] = carbon-11; [^18^F] = fluorine-18; Aβ = amyloid-β; DLB = dementia with Lewy bodies; HC(s) = healthy control(s); GBA1 = glucosylceramidase beta 1; MSA = multiple system atrophy; MSA-C = multiple system atrophy with predominant cerebellar ataxia; PD = Parkinson’s disease; PET = positron emission tomography. ACI-12589, C05-05, SPAL-T-06, MODAG-005, and HY-2-15 are conventional radiotracer development codes.

## Data Availability

No new data were created or analysed in this study. Data sharing is not applicable to this article.

## Abbreviations

αSyn-SAA	α-Synuclein seed amplification assay
Aβ	Amyloid-β
AADC	Aromatic L-amino acid decarboxylase
AI	Artificial intelligence
BBB	Blood–brain barrier
BP_ND_	Binding potential relative to the non-displaceable compartment
CBS	Corticobasal syndrome
CNN	Convolutional neural network
COMT	Catechol-O-methyltransferase
CSF	Cerebrospinal fluid
CT	Computed tomography
D1/D5	Dopamine D1/D5 receptor subtypes
D2/D3	Dopamine D2/D3 receptor subtypes
DAT	Dopamine transporter
DLB	Dementia with Lewy bodies
DMFP	Desmethoxyfallypride
DTBZ	Dihydrotetrabenazine
EANM/SNMMI	European Association of Nuclear Medicine/Society of Nuclear Medicine and Molecular Imaging
FDG	Fluorodeoxyglucose
FDOPA	6-Fluoro-L-DOPA
FMT	6-Fluoro-L-m-tyrosine
GBA1	Glucosylceramidase beta 1
GPCR	G protein-coupled receptor
HC	Healthy control
IBZM	Iodobenzamide
iRBD	Isolated rapid eye movement sleep behaviour disorder
K_i_	Influx constant
L-DOPA	L-3,4-Dihydroxyphenylalanine
LAT1	Large neutral amino acid transporter type 1
LRRK2	Leucine-rich repeat kinase 2
MAO	Monoamine oxidase
MIBG	Metaiodobenzylguanidine
ML	Machine learning
MRI	Magnetic resonance imaging
MSA	Multiple system atrophy
MSA-C	Multiple system atrophy with predominant cerebellar ataxia
MSA-P	Multiple system atrophy with predominant parkinsonism
NET	Noradrenaline transporter
NSD-ISS	Neuronal α-synuclein disease integrated staging system
PD	Parkinson’s disease
PET	Positron emission tomography
PINK1	PTEN-induced kinase 1
PRKN	Parkin RBR E3 ubiquitin protein ligase
PSP	Progressive supranuclear palsy
R_1_	Relative tracer-delivery parameter
ROI	Region of interest
SBR	Specific binding ratio
SERT	Serotonin transporter
SNpc	Substantia nigra pars compacta
SPECT	Single-photon emission computed tomography
SUVR	Standardised uptake value ratio
SWEDD	Scans without evidence of dopaminergic deficit
SynNeurGe	α-Synuclein pathology, neurodegeneration, and genetics
TH	Tyrosine hydroxylase
TM	Transmembrane
VMAT2	Vesicular monoamine transporter type 2
VOI	Volume of interest
